# Enhanced Epigenetic Modulation via mRNA-Encapsulated Lipid Nanoparticles Enables Targeted Anti-inflammatory Control

**DOI:** 10.1021/acssynbio.5c00188

**Published:** 2026-01-15

**Authors:** Tahere Mokhtari, Mohammad N. Taheri, Sarah Akhlaghi, Armin Aryannejad, Yuda Xiang, Vineet Mahajan, Kamyar Keshavarz, Amirreza Kiani, Samantha Yang, Samuel LoPresti, Ryan LeGraw, Kathryn A. Whitehead, Samira Kiani

**Affiliations:** † Division of Experimental Pathology, Department of Pathology, 12317 University of Pittsburgh School of Medicine, Pittsburgh, Pennsylvania 15261, United States; ‡ Pittsburgh Liver Research Center, 12317 University of Pittsburgh School of Medicine, Pittsburgh, Pennsylvania 15261, United States; § Department of Bioengineering, 6614 University of Pittsburgh, Pittsburgh, Pennsylvania 15213, United States; ∥ Department of Electrical and Computer Engineering, 7938 University of Toronto, Toronto, Ontario M5S 3G4, Canada; ⊥ Department of Chemical Engineering, 6612 Carnegie Mellon University, Pittsburgh, Pennsylvania 15213, United States; # School of Medicine, Tsinghua Medicine, Tsinghua University, Beijing 100084, China; ∇ GenexGen Inc., 169 East Portola Avenue, Los Altos, California 94022, United States; ○ HeXembio Inc., 877 Francisco Street, Los Angeles, California 90017, United States

**Keywords:** Zinc finger transcriptional repressors, Immunomodulation, Lipid nanoparticles, Myd88, Epigenetic engineering, Inflammatory diseases

## Abstract

Temporal transcriptional modulation of immune-related genes offers powerful therapeutic potential for treating inflammatory diseases. Here we introduce an enhanced zinc finger (ZF)-based transcriptional repressor delivered via lipid nanoparticles for controlling immune signaling pathways *in vivo*. By targeting Myd88, an essential adaptor molecule involved in immunity, our system demonstrates therapeutic efficacy against septicemia in C57BL/6J mice and improves repeated AAV administration by reducing antibody responses. This epigenetic engineering approach provides a platform for safe and efficient immunomodulation applicable across diseases caused by imbalanced inflammatory responses.

## Graphical abstract

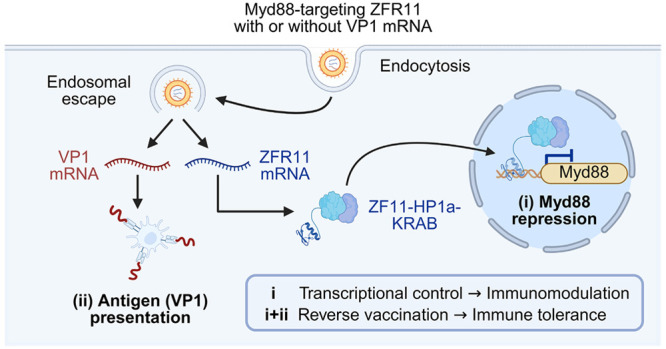

Overactive immune responses pose significant challenges in various inflammatory diseases, often leading to severe tissue damage, organ dysfunction, and mortality.^1^ This phenomenon, termed “cytokine storm”, manifests through uncontrolled release of pro-inflammatory mediators and has been implicated in conditions ranging from acute respiratory distress syndrome (ARDS) to sepsis.^2,3^ Current therapeutic approaches rely largely on broad-spectrum immunosuppressants, which can leave patients vulnerable to opportunistic infections and lead to significant adverse effects.^4–7^

Beyond acute inflammatory conditions, immune responses hamper the success of gene therapies, particularly for those exploiting adeno-associated virus (AAV) delivery.^8^ Pre-existing humoral immunity against AAV excludes many patients from trials and compromises treatment outcomes.^9,10^ Several factors influence viral vector immunogenicity, including capsid serotype, vector DNA, target tissue inflammatory state, and host immunity status.^11,12^ While various strategies attempt to regulate immune responses against AAV vectors, existing approaches face limitations in overcoming pre-existing immunity and enabling vector redosing.^13–16^

Myd88, an essential adaptor for Toll-like receptors (TLRs) and upstream regulator of NF-κB signaling, represents a promising target for addressing both hyperinflammatory conditions and gene therapy challenges.^17^ MyD88 signaling has been directly implicated in forming immune responses against AAV capsid, which leads to loss of transgene expression and limits effective administration of the virus.^18^ Furthermore, MyD88 signaling is essential for survival against viral infections, making it an attractive target for modulating both innate and adaptive immunity to viral vectors.^19^ Traditional approaches for targeting *Myd88*, such as small molecule inhibitors, have been explored but suffer from lack of specificity and proper tissue penetrance *in vivo*.^20,21^

While our previous work demonstrated that CRISPR-based epigenetic downregulation of *Myd88* delivered via AAVs can effectively dampen immune responses,^22^ this approach faces limitations including potential immunogenicity of CRISPR and AAV components, lack of transient control, and challenges in scalability. To address these challenges, we introduce a novel method for synthetic immunomodulation using mRNA-based zinc finger transcriptional repression targeting *Myd88*. Our strategy involves zinc finger directed epigenetic modulation, allowing precise and reversible control over gene expression. Zinc finger proteins offer several advantages over other epigenetic-editing technologies, including low immunogenicity, smaller size for easier packaging and delivery, and high DNA binding specificity.^23,24^ To avoid complexities associated with viral vehicles, we deliver the zinc finger-based epigenetic repressor mRNA via lipid nanoparticles (LNPs), providing an attractive platform for achieving transient gene regulation while offering potential for scale-up.^25^

## Zinc Finger-Mediated Repression with HP1a-KRAB Efficiently Downregulates *Myd88 In Vitro*

In our previous studies, we established CRISPR-based epigenetic repressors using two methods: direct fusion of modulatory elements to a catalytically inactive Cas9 and an indirect approach utilizing aptamers to recruit MS2-fused transcriptional repressors to the CRISPR complex.^22,26^ Buildingon our prior findings, we sought to develop a zinc finger (ZF)-tethered *Myd88*-targeting repressor with enhanced efficacy and specificity. We designed a panel of 16 zinc finger sequences targeting distinct regions of the *Myd88* promoter and evaluated their repressive capacity when fused to HP1a-KRAB. Through systematic screening in mouse neuroblastoma (N2A) cells and quantitative real-time polymerase chain reaction (qRT-PCR), we identified zinc finger-effector 11 (ZFR11) as the most potent repressor of *Myd88* expression (Figure S1A–C and Table S1).

We next compared the performance of ZFR11 with our previously optimized CRISPR-based system, which utilizes a 14-nucleotide truncated guide RNA, Cas9 nuclease, and MS2-HP1a-KRAB.

qRT-PCR analysis revealed that ZFR11 achieved *Myd88* repression comparable to that achieved with an enhanced aptamer-mediated CRISPR repressor (Figure S1D). These findings reveal the *in vitro* functionality of the ZFR11 and underscore its potential as a highly efficient tool for modulating *Myd88* expression, offering an alternative to CRISPR-based approaches.

## 306O_10_ LNP-Mediated Delivery Enables Effective Transfection of Diverse Immune Cell Populations *In Vitro* and *In Vivo*

To enhance the efficiency of *Myd88* repression and enable transient immunomodulation, we constructed epigenetic modulators in the form of mRNA, which were subsequently delivered into diverse cells via LNPs. LNP-mediated mRNA delivery provides several advantages over viral and other nonviral delivery platforms. Most importantly, mRNAs are biodegradable and short-lived, thus allowing temporary expression and circumventing genome integration.^25,27^ Compared to viral vectors, lipid-mediated delivery is superior in terms of larger payload capacity and ease of preparation in addition to its lower immunogenicity, toxicity, and higher safety profiles.^28,29^ Extensive literature has documented the therapeutic potential of LNP-mediated delivery platforms in immunotherapy.^30–32^ After screening a library of ionizable LNPs, we identified 306O_10_ as a promising candidate based on its previously demonstrated efficacy in mRNA-based immunotherapy and CRISPR/Cas9 delivery.^33–37^ 306O_10_ LNPs demonstrated lower immunogenicity compared to other formulations and exhibited tropism toward immune cell populations in the spleen and liver, key sites for modulating systemic immune responses.^38^ To maximize therapeutic efficacy, we incorporated DOPS (1,2-dioleoyl-*sn*-glycero-3-phospho-l-serine) as a helper lipid in our LNP formulation, which has been shown to enhance spleen targeting.^38^ Characterization of our LNP formulations confirmed particle size (103–132 nm), distribution polydispersity index (PDI < 0.25), and RNA encapsulation efficiency (52–62%) across all constructs (Figure S2 and Table S2).

Initial *in vitro* studies in RAW 264.7 macrophages confirmed efficient 306O_10_-mediated mRNA delivery, evidenced by robust mCherry expression 24 h post-transfection (Figure S3A). To assess *in vivo* performance and biodistribution, we administered GFP mRNA-loaded particles to C57BL/6 mice via retro-orbital (RO) or tail vein (TV) injection (Figure S3B). Four hours following LNP delivery, we collected lung, liver, and spleen and assessed the transcript levels of GFP. Quantitative RT-PCR analysis revealed widespread GFP mRNA distribution across lung, liver, and spleen tissues, with the RO route yielding the highest transfection efficiency (Figure S3C). To further validate these GFP qRT-PCR results at the protein level, immunohistochemical (IHC) staining for GFP was conducted on tissue sections from the spleen, liver, and lungs of mice injected with GFP mRNA LNPs. In the lungs, immunostaining was detected in the bronchiolar epithelium, type II pneumocytes, intra-alveolar and intravascular histiocytes and neutrophils, as well as the vascular intima, signifying LNP uptake into these cell populations. Similarly, immunostaining in the liver occurred ubiquitously in all hepatocytes, bile ducts, and intravascular inflammatory cells, with little to no staining present in connective tissues. In the spleen, GFP staining was largely restricted to the erythrocytes and macrophages within the red pulp, with occasional staining of germinal centers (Figure S3D). Quantitative analysis of GFP immunostaining intensity confirmed the mRNA expression patterns, with retro-orbital administration yielding higher protein expression compared to tail vein injection across all tissues examined (Figure S3E). This protein-level validation corroborates our qRT-PCR findings and demonstrates efficient translation of the delivered mRNA. The cell type-specific GFP expression pattern observed through immunostaining further confirms that 306O_10_ LNPs successfully target and deliver functional cargo to distinct cell populations within these tissues.

Previous studies have characterized the tropism of 306O_10_ in placental tissue, demonstrating broad distribution across diverse immune and nonimmune cell populations.^39^ To further define the cell type-specific biodistribution of 306O_10_ LNPs, we investigated the cellular uptake patterns in the liver and spleen.

In the liver, GFP-306O_10_ uptake was evaluated in primary cultures of liver sinusoidal endothelial cells (LSECs), Kupffer cells, and hepatocytes. Quantitative RT-PCR analysis of GFP transcript levels at 48 h post-transfection revealed substantial uptake in LSECs, Kupffer cells, and hepatocytes (Figure S4A).

The immunological targeting profile of 306O_10_ in the spleen was examined using the experimental design outlined in Figure S4B. GFP mRNA-encapsulated LNPs were administered via intravenous injection to C57BL/6J mice. Spleen tissues were harvested 4 h post-administration for subsequent immune cell isolation and quantitative analyses. Magnetic-activated cell sorting (MACS) was employed to isolate CD19^+^, CD11c^+^ and CD11c^–^ populations. LNP uptake within each subset was then quantified by qRT-PCR measurement of GFP mRNA (Figure S4C). GFP protein expression in the spleen was further confirmed via flow cytometry, which demonstrated a rightward shift in GFP fluorescence signal intensity relative to unstained and uninjected control samples (Figure S4D). Quantitative flow cytometry analysis verified the transfection efficiency of 306O_10_ LNPs for cargo delivery to CD19^+^ (7.2% GFP^+^), CD11b^+^ (24% GFP^+^), and CD11c^+^ (2% GFP^+^) cell populations in the spleen compared to matching unstained and uninjected controls (Figure S4E,F). These findings highlight the versatility of 306O_10_ LNPs for targeting diverse immune cell populations, a critical feature for immunomodulatory applications.

## ZF-Based Repression of the *Myd88* Locus Achieves Efficient Immunomodulation under Both Homeostatic and LPS-Induced Inflammatory Conditions

Having validated our ZF-based repressor *in vitro* and optimized its delivery via 306O_10_ LNPs, we set out to determine whether systemic administration of ZFR11–306O_10_ could effectively repress endogenous *Myd88* expression in wild-type C57BL/6 mice. To test this, an experiment was devised as illustrated in Figure S5A. Mice received a single intravenous injection of either ZFR11-mCherry-306O_10_, mCherry-306O_10_, or PBS (no LNP). Six hours post-administration, euthanasia was performed, and blood, lung, and spleen were harvested for RT-qPCR measurement of *Myd88* mRNA levels. We observed *Myd88* repression in ZFR11-mCherry-306O_10_ treatment group across multiple tissues, with transcript levels reduced by 50% in the spleen, 46% in blood, and 16% in lung compared to mCherry-306O_10_ controls (Figure S5B). Additionally, *Myd88* repression was accompanied by a concomitant downregulation of key downstream inflammatory mediators, such as *Icam-1*, *Tnf-α*, *Ncf*, *Il6*, *Ifn-α*, *Ifn-β*, *Ifn-γ*, *Il-1β*, and *Stat4* (Figure S5C).

We next investigated whether LNP-mediated delivery of the *Myd88* repressor could modulate inflammatory responses in lipopolysaccharide (LPS)-induced septicemia, a clinically relevant model associated with elevated *Myd88* expression.^40–42^ Septicemia is particularly well suited for evaluating *Myd88*-targeted interventions, as it remains a significant global health burden with limited therapeutic options due to its complex pathophysiology driven by dysregulated inflammatory cascades.^43,44^

C57BL/6 mice received intraperitoneal LPS injection followed by intravenous administration of ZFR11–306O_10_ 2 h later (Figure 1A). Remarkably, 24 h post-LNP treatment, we observed robust *Myd88* repression in blood (79%), lung (58%), and liver (22%) compared to PBS-treated controls (Figure 1B). This repression effectively prevented the LPS-induced upregulation of multiple inflammatory mediators downstream of *Myd88* signaling, including *Icam-1*, *Tnf-α*, *Ncf*, *Il6*, *Ifn-α*, *Ifn-β*, *Ifn-γ*, and *Stat4* (Figure 1C–E). In addition, quantitative chemiluminescent ELISA analysis of plasma cytokines revealed a trend toward reduced cytokine levels in *Myd88*-repressed mice, further supporting the anti-inflammatory effects of ZF-based *Myd88* repression (Figure 1F). Collectively, these results demonstrate that LNP-mediated delivery of ZFR11 can effectively modulate *Myd88* expression under both homeostatic and inflammatory conditions, establishing proof of concept for this synthetic biology approach to immunomodulation.

**Figure 1:**
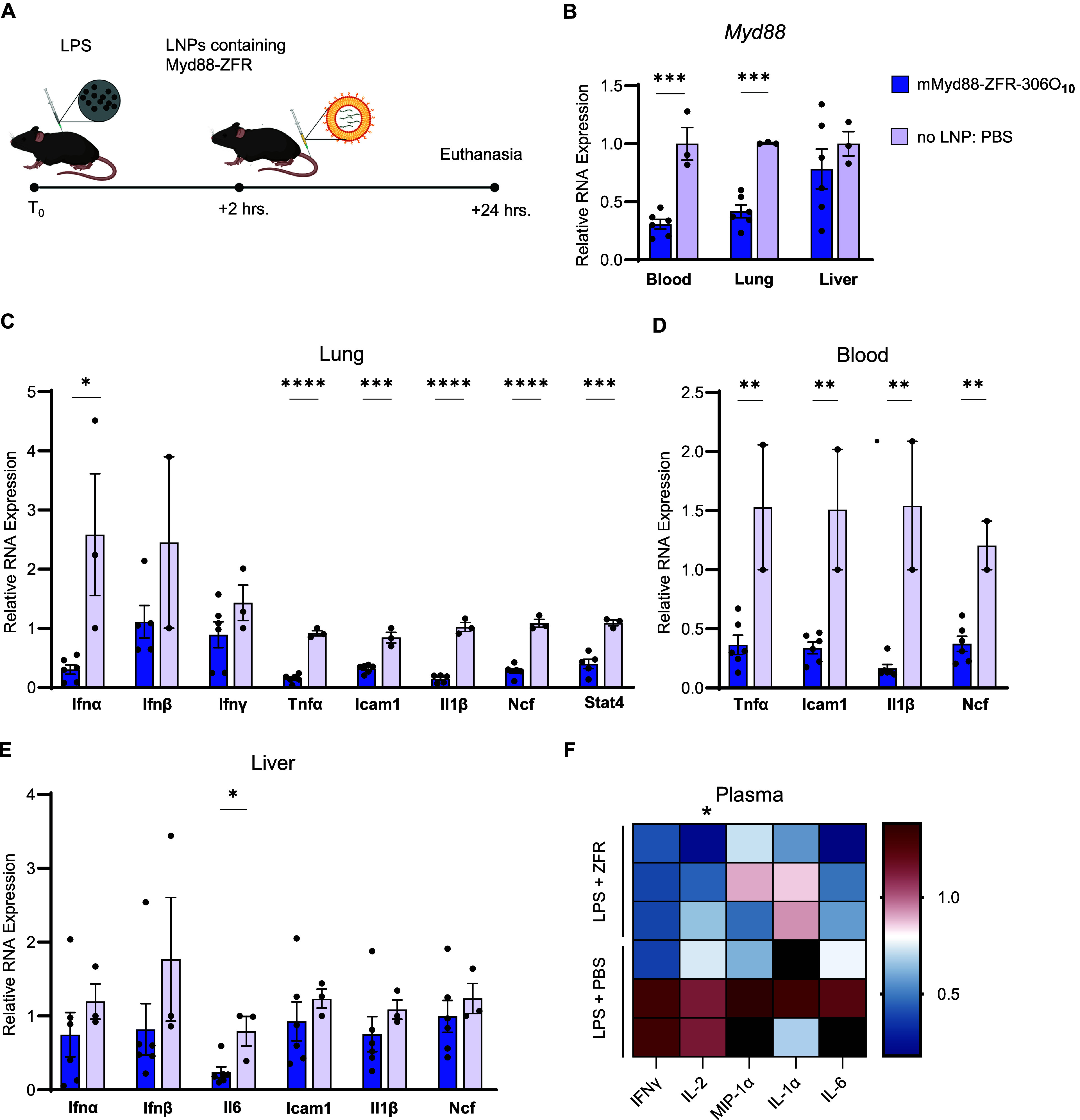
Delivery of lipid nanoparticles carrying mRNA encoding *Myd88*-targeting zinc finger enables immunomodulation during LPS-induced inflammation. (A) Schematic representation of experimental design. C57BL/6 mice received intraperitoneal LPS injection (2.5 mg kg^–1^) followed by tail vein administration of either ZFR11–306O_10_ or PBS (no LNP control) 2 h later. Tissues were collected 24 h post-LNP administration. (B) qRT-PCR analysis of *Myd88* expression in blood, lung, and liver tissues following LPS challenge and zinc finger-mediated repression (*N* = 6 mice for ZFR11–306O_10_ group, *N* = 3 for PBS control group). Data are presented as mean ± SEM. (C) qRT-PCR analysis of inflammatory gene expression (*Ifn-α*, *Ifn- β*, *Ifn-γ*, *Icam-1*, *Ncf*, *Tnf-a*, *Il-1β*, and *Stat4*) in lung. Expression levels were normalized to PBS-treated control mice (*N* = 6 for ZFR11–306O_10_ group, *N* = 3 for PBS group). Data are presented as mean ± SEM (D) qRT-PCR analysis of inflammatory gene expression (*Tnf-a*, *Icam-1, Il-1β*, and *Ncf*) in blood. Expression levels were normalized to PBS-treated control mice (*N* = 6 for ZFR11–306O_10_ group, *N* = 3 for PBS group). Data are presented as mean ± SEM (E) qRT-PCR analysis of inflammatory gene expression (*Ifn-α*, *Ifn-β*, *Il6*, *Icam-1*, *Il-1β*, and *Ncf*) in liver. Expression levels were normalized to PBS-treated control mice (*N* = 6 for ZFR11- 306O_10_ group, *N* = 3 for PBS group). Data are presented as mean ± SEM (F) Measurement of a panel of inflammatory cytokines in plasma using a multiplex-ELISA assay; values are displayed in the heatmap as relative measured concentration (pg/mL) compared to PBS-treated control mice.: IFNγ (*N* = 2 per group), IL2 (*N* = 3 for ZFR11–306O_10_, *N* = 2 for PBS), MIP-1a (*N* = 3 for ZFR11–306O_10_, *N* = 2 for PBS), IL-1a (*N* = 3 for ZFR11–306O_10_, *N* = 2 for PBS), and IL-6 (*N* = 3 for ZFR11–306O_10_, *N* = 2 for PBS). IFNγ, interferon gamma; IL, interleukin; MIP, macrophage inflammatory protein. Statistical analysis was performed using the unpaired parametric *t* test. *P ≤ 0.05 was considered statistically significant.

To provide a comparative assessment of our zinc finger approach against established CRISPR-based systems, we conducted parallel studies using our previously optimized dCas9-KRAB-MeCP2 system delivered via 306O_10_ LNPs under identical experimental conditions in the LPS-induced inflammation model (Figure S6A). The CRISPR-based dCas9 approach (dCas9-KRAB-MeCP2 mRNA at 1.6 mg/kg;*Myd88*-targeting gRNA at 0.4 mg/kg) achieved only modest *Myd88* repression of 25% and 16% in bone marrow and blood, respectively, at 24 h post-treatment, with no detectable repression in spleen, liver, or lung tissues (Figure S6B,C). To investigate whether extended exposure duration might enhance efficacy, tissues were additionally harvested at 72 h post-treatment (Figure S6D). While this extended time frame showed a trend toward 45% *Myd88* reduction in liver tissue, repression remained absent in spleen and lung tissues (Figure S6E,F). These comparative findings suggest that zinc finger-based transcriptional repression may offer advantages over CRISPR-mediated approaches for achieving robust *Myd88* suppression in this acute inflammatory context, potentially due to differences in construct size, immunogenicity, and delivery efficiency.

## ZFR11 Functions as an Antiadjuvant to Modulate AAV-Directed Humoral Immunity and Enhance the Efficiency of Viral-Based Gene Delivery

To mount an effective adaptive immune response, antigen-presenting cells must activate T cells through both antigen presentation and costimulatory signals at the immunological synapse. This principle is leveraged in vaccines through the use of adjuvants, which enhance immune activation. A critical component of these stimulating signals is inflammatory cytokine production, which is regulated by NF-κB signaling, with Myd88 serving as a key upstream mediator. We predicted that selective *Myd88* repression during antigen exposure could function as an “anti-adjuvant”, attenuating inflammation at the immunological synapse and preventing immune activation. We therefore investigated whether our *Myd88* repressor could regulate humoral immunity against AAV vectors in preimmunized hosts, addressing a major challenge in AAV-based gene therapy redosing. This anti-adjuvant strategy has shown promise across multipletherapeutic areas, including allergy immunotherapy, transplant tolerance, and autoimmune disease treatment.^45–48^

To investigate the effect of *Myd88* repression in hosts with pre-existing anti-AAV antibodies, we established an AAV2-pre-exposed mouse model (Figure 2A) using AAV2, an FDA-approved gene therapy vector. C57BL/6J mice received two retro-orbital (RO) injections of AAV2-GFP to induce pre-existing immunity. We developed a reverse vaccination strategy using LNP-306O_10_-encapsulated mRNA encoding three components: ZFR11 *Myd88* suppressor (anti-adjuvant), VP1 (AAV2 capsid antigen), and mCherry. Prior to *in vivo* experiments, we validated expression of the reverse vaccine components. Immunofluorescence analysis confirmed that 306O_10_ LNPs successfully delivered both VP1 and ZFR11 repressor mRNAs, resulting in robust protein expression in RAW 264.7 macrophages 24 h post-transfection (Figure S7). Having confirmed functional protein expression of both vaccine components, we proceeded with *in vivo* AAV pre-immunization studies.

**Figure 2:**
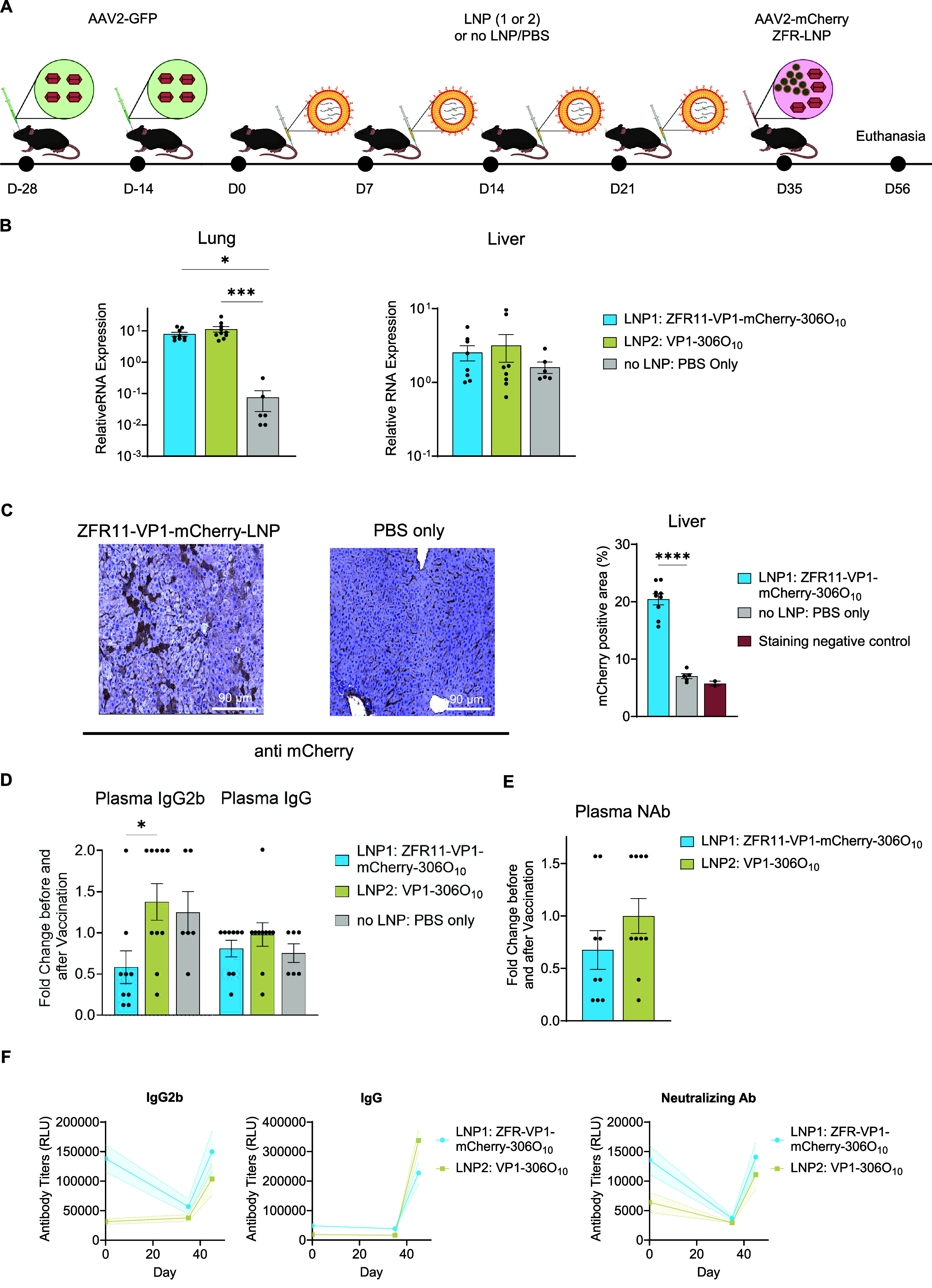
Co-delivery of *Myd88* repressor ZFR11 and gene therapy related antigen modulates humoral immunity in AAV preimmunized mice. (A) Schematic representation of experimental design. C57BL/6J mice received two retro-orbital injections of AAV2-GFP (5 × 10^11^ GC) 2 weeks apart. Two weeks later, mice were administered weekly doses of LNP-encapsulated mRNA (0.7 mg kg^–1^) encoding either ZFR11-VP1-mCherry (*N* = 10), VP1 only (*N* = 10), or no LNP/PBS control (*N* = 6) for 4 weeks. Two weeks after the final vaccination, mice received a single intravenous coinjection of AAV2-mCherry (5 × 10^11^ GC) and ZFR11-LNP (0.7 mg kg^–1^). Analyses were performed 3 weeks post-AAV2 challenge. (B) qRT-PCR analysis of *mCherry* expression in liver and lung tissues (*N* = 8–10 mice per treatment group, *N* = 6 for PBS). Data are presented as mean ± SEM Statistical analysis was performed using one-way ANOVA followed by Dunnett’s multiple comparisons test. *P ≤ 0.05 was considered statistically significant (C) Left: Representative immunohistochemical staining for mCherry in liver sections. Right: Quantification of mCherry staining intensity (ZFR11-VP1-mCherry-306O_10_: *N* = 9, PBS: *N* = 5, staining negative control/mCherry uninjected: *N* = 2). Data are presented as mean ± SEM Statistical analysis was performed using one-way ANOVA followed by Dunnett’s multiple comparisons test. *P ≤ 0.05 was considered statistically significant. (D) ELISA measurement of plasma anti-AAV2 total IgG and IgG2b levels. Results shown as fold change in optical density at day 35 (2 weeks postvaccination) relative to day 0 (prevaccination baseline) (*N* = 9–10 per treatment group, *N* = 6 for PBS). Statistical analysis was performed using one-way ANOVA followed by Dunnett’s multiple comparisons test. *P ≤ 0.05 was considered significant. (E) ELISA measurement of serum anti-AAV2 neutralizing antibody levels showing a 32% reduction in the ZFR11-VP1-mCherry group compared to VP1 control at day 35 (2 weeks postvaccination) relative to day 0 (prevaccination baseline) (*N* = 9–10 mice per treatment group, *N* = 6 mice for PBS). Statistical analysis was performed using the unpaired parametric *t* test. *P ≤ 0.05 was considered significant. (F) Time-point analysis of plasma total IgG, IgG2b, and neutralizing antibodies titers in Relative Light Units (RLU) at day 0 (prevaccination baseline), day 35 (2 weeks postvaccination), and day 45 (10 days after AAV2-mCherry rechallenge) (*N* = 9–10 per treatment group).

Mice were divided into three groups (*N* = 10 per group) receiving four weekly doses of: ZFR11-VP1-mCherry (group 1), VP1 alone (group 2), or PBS control (group 3). Two weeks after the final vaccination, all mice received a single intravenous coinjection of AAV2-mCherry and ZFR11–306O_10_. Analysis was performed 3 weeks post-AAV2-mCherry challenge, followed by collection of blood and tissue samples for downstream processing.

We first assessed whether vaccination with *Myd88* repressor could influence the efficiency of AAV-mediated transgene delivery, mimicking clinical scenarios requiring therapeutic gene delivery in AAV pre-exposed populations. Quantitative RT-qPCR analysis identified significantly elevated mCherry (AAV cargo transgene) transcript levels in lung tissues from mice receiving ZFR11-VP1-mCherry or VP1 alone vaccinations compared with PBS controls (∼100-fold and ∼150-fold, respectively) (Figure 2B). A similar trend was observed in liver tissue, albeit with more modest increases (∼1.6-fold and ∼2-fold, respectively). In agreement with earlier qRT-PCR results, quantification of nonfluorescent IHC images confirmed an ∼3-fold increase in mCherry protein expression in liver sections from ZFR11-VP1-mCherry-vaccinated mice relative to PBS control (Figure 2C).

Next, we investigated whether treatment with ZFR11-VP1-mCherry-306O_10_ LNPs could attenuate AAV-specific humoral responses by measuring both immunoglobulin G (IgG) levels and neutralizing antibody titers. Anti-AAV2 IgG and IgG2b levels were quantified in plasma by enzyme-linked immunosorbent assay (ELISA). At day 35 (post-vaccination), we observed that ZFR11-VP1-mCherry vaccination significantly reduced anti-AAV2 IgG2b levels by 58% compared with VP1 controls (Figure 2D). This group also showed a trend toward lower total IgG, with levels 18% below VP1 controls (Figure 2D). Additionally, neutralizing antibody levels were reduced by 32% in ZFR11-VP1-mCherry vaccinated animals compared with VP1 controls before (day 0) and after vaccination (day 35) (Figure 2E). While ZFR11-VP1-mCherry vaccination initially reduced IgG2b and neutralizing antibody levels (comparing days 0 and 35, prior to and post vaccination), this tolerance effect was transient. AAV2-mCherry rechallenge at 2 weeks post-injection (day 45) restored antibody levels to those observed following initial AAV2-GFP administration (Figure 2F). Notably, the therapeutic intervention prevented further upregulation beyond baseline, representing this strategy’s efficacy in maintaining stable antibody responses.^44^ In addition, safety assessment throughout the study revealed no adverse effects associated with repeated LNP administration (Figure S8). All treatment groups maintained body weight (Figure S8A), and plasma biochemical markers of hepatic function revealed no elevation in alanine aminotransferase (ALT) or bile acids in groups receiving vaccination with mRNAs encoding ZFR11-VP1-mCherry or VP1 antigen alone, compared with PBS control (Figure S8B), confirming that the current dose of 306O_10_ LNPs was well-tolerated.

These results underscore the immunomodulatory effects of ZFR11-VP1-mCherry as an anti-inflammatory agent, suggesting that it can modify established immune responses even in the context of pre-existing immunity, although further optimization of dose, timing, and dosing frequency is needed (Figure S2F).

Our work establishes a novel epigenetic editor for targeted immunomodulation using zinc finger-based transcriptional repressors. Exploiting this modality, we demonstrated efficient repression of endogenous Myd88 both *in vitro* and *in vivo*, effectively dampening inflammatory responses during LPS-induced model of septicemia and enhancing outcomes in AAV gene therapy applications. This strategy addresses key limitations of current immunomodulatory approaches by providing precise and transient control over immune responses.

Our approach, which uses an effective LNP delivery system to deliver zinc finger repressor-encoding mRNAs, represents a significant advance over existing methods. Unlike viral vectors or small molecule inhibitors, our work combines the specificity of zinc finger proteins with the versatility of 306O_10_ lipid nanoparticles to enable transient immunomodulation of immune cell populations.^38,39^ By incorporating DOPS into the 306O_10_ formulation, as previously demonstrated by LoPresti et al. for spleen-targeted delivery,^38^ we achieved organ-selective immunomodulation. This anionic lipid promotes preferential uptake by splenic immune cells while reducing hepatic delivery, thereby concentrating the therapeutic effect in the primary site of systemic immune regulation while minimizing potential liver-associated side effects. In addition, the scalability and relatively straightforward manufacturing of LNP-based delivery supports clinical translation, whereas viral vectors often face production and scalibility challenges.

Our data demonstrate two distinct therapeutic applications of this platform. In the context of acute inflammation, ZFR11-mediated *Myd88* repression effectively reduces the cascade of inflammatory signaling typically associated with septicemia. This suggests potential applications in inflammatory conditions where precise, temporary immune suppression is desirable.

In the context of AAV gene therapy, our findings usher in a new era for mitigating pre-existing immunity. The ability to temporarily suppress anti-AAV antibody production through *Myd88* repression provides a viable solution to one of the field’s most significant challenges. While the effect is transient, this window of reduced immunity may be sufficient to enable successful vector readministration.

Future applications of this platform may benefit from additional optimizations. Alternative dosing schedules, modified LNP formulations, or combinations with existing immunomodulatory approaches could extend the duration of effect. Additionally, the modularity of our system allows for targeting of immune regulators beyond *Myd88*, potentially expanding its utility across diverse immunological conditions. Further investigations can unveil the mechanisms underlying the observed immunomodulatory effects and explore broader applicability across additional disease models

In conclusion, our findings highlight the potential of combining precisely tuned gene regulation modalities with enhanced mRNA delivery strategies to address diseases characterized by dysregulated inflammatory pathways. Our study opens new possibilities for achieving precise, safe, and effective modulation of immune responses across a broad spectrum of diseases.

## Methods

### Zinc Finger *Myd88* Repressor Fusion DNA Constructs

A panel of 16 zinc finger (ZF) sequences were designed to target specific regions within the mouse *Myd88* promoter, spanning a 300 bp region upstream of the transcription start site (TSS). These target sites were selected through *in silico* analysis by Sigma, using their proprietary algorithm that suggests potential binding sites in gene promoters for zinc finger-based repressors with higher efficiency and minimal off-target effects. Each zinc finger repressor (ZFR) targets a distinct 28 bp sequence, with ZFRs 1–8 targeting the forward strand and ZFRs 9–16 targeting the reverse strand (Figure S1A and Table S1). ZFR11 was selected as the lead candidate based on its optimal binding specificity score (19.96) and superior *Myd88* repression efficiency (>70% in N2A cells). Binding specificity scores were calculated using Sigma-Aldrich’s proprietary computational algorithm, which performs genome-wide off-target analysis using position weight matrices to evaluate predicted binding affinity and specificity. The specificity score integrates both the strength of on-target binding and the absence of predicted off-target sites across the genome. Among the 16 candidates, ZFR11 achieved the computational specificity score of 19.96. Higher scores indicate stronger predicted binding affinity and specificity for the target sequence, with reduced potential for off-target binding. DNA templates were synthesized by Integrated DNA Technologies (IDT) and quality was verified by Sanger sequencing and agarose gel electrophoresis. The 16 ZFRs were constructed through a multistep cloning process. First, the repressive domains (HP1a and KRAB) were PCR-amplified from previously constructed vectors described in our earlier work.^22^ These domains were then fused via overlap extension PCR to generate the HP1a-KRAB DNA fragment. The resulting fragment was inserted into an L1L2 entry backbone using BsaI-based Golden Gate cloning (NEBridge Golden Gate Assembly Kit, Cat. No. E1601S). A library of 16 distinct zinc finger DNA fragments, each targeting different regions of the *Myd88* promoter, was then cloned upstream of the HP1a-KRAB using In-Fusion cloning technology (Takara Bio’s In-Fusion Snap Assembly Master Mix, Cat. No. 638948). The final expression constructs were assembled through a three-fragment Gateway recombination reaction (ThermoFisher Scientific, Cat. No. 12538120) utilizing L4R1-CAG (promoter), L1L2-ZF-HP1a-KRAB (repressor), and R1R2-LVGTW3 (terminator) constructs. This approach generated 16 distinct ZF repressor DNA constructs. Complete sequences of all zinc finger arrays and the ZFR1-HP1a-Krab fusion DNA fragment are provided in Table S3.

### Optimization of ZF-Based *Myd88* Repressor for Enhanced Efficacy and Reduced Immunogenicity

To maximize the therapeutic potential of our ZF-based *Myd88* repressor while minimizing undesired immune responses, we implemented a series of strategic design optimizations. These modifications encompassed both the mRNA component and the choice of epigenetic modulator. For the mRNA component, we incorporated N1-methyl pseudouridine nucleoside modifications at 100% substitution ratio and employed a uridine depletion strategy by replacing third-position uridines with cytosines where synonymous codons allowed, achieving approximately 40% uridine reduction. N1-methyl pseudouridine has been shown to enhance mRNA stability and reduce recognition by pattern recognition receptors, thereby attenuating innate immune activation.^49–52^ Uridine depletion further contributes to reduced immunogenicity by minimizing uridine-rich sequences that can trigger Toll-like receptor activation.^53^

Using Geneious software, we optimized the Zinc finger nucleic acid sequences for mammalian codon usage, a strategy that has been demonstrated to improve translational efficiency and potentially reduce immunogenicity by mimicking endogenous mRNA characteristics.^54,55^ To eliminate double-stranded RNA (dsRNA) contaminants, which are potent activators of innate immune responses, we employed a cellulose-based dsRNA removal process in our mRNA preparation protocol.^56,57^

The selection of zinc finger proteins as our epigenetic modulator was informed by their favorable immunological profile and structural characteristics. Compared to other gene-editing technologies such as CRISPR-Cas9, zinc finger proteins exhibit lower immunogenicity and have a more compact structure, potentially facilitating delivery and reducing the likelihood of eliciting an immune response.^58^

These multifaceted optimizations were designed to synergistically enhance the efficacy of our ZF-based *Myd88* repressor while minimizing its potential to trigger undesired immune responses, thereby improving its therapeutic index and translational potential.

### mRNA and Lipids

The following mRNAs and reagents were used for nanoparticle formulation: CleanCap modified mRNAs (encoding GFP, mCherry, ZFR11, and VP1) from TriLink Biotechnologies; Cholesterol from Sigma-Aldrich (catalog number C8667, ≥99% pure, extracted from sheep wool); and phospholipids from Avanti Polar Lipids including DOPS (sodium 1,2-dioleoyl-*sn*-glycero-3-phospho-l-serine, catalog number 840035P) and C14-PEG2000 (ammonium 1,2-dimyristoyl-*sn*-glycero-3-phosphoethanolamine-*N*-[methoxy­(polyethylene glycol)-2000], catalog number 880150P).

### mRNA Synthesis

All mRNAs were synthesized by TriLink Biotechnologies using their CleanCap modified mRNA synthesis service. The mRNA was synthesized within specially designed plasmids containing T7 promoter, proprietary 5′ and 3′ UTRs, and a 120 nucleotide poly­(A) tail incorporated via PCR. Critically, standard UTP was replaced with *N1*-methylpseudouridine-5′-triphosphate at 100% substitution ratio to reduce immunogenicity while maintaining translation efficiency. 5′-triphosphate removal was performed via phosphatase treatment to further reduce innate immune response activation.

### mRNA Purification and Quality Control

Purification was performed using silica membrane-based methods with cellulose-based double-stranded RNA removal. Quality control metrics confirmed A260/A280 ratios of 1.9–2.2 and agarose gel electrophoresis showed single bands without degradation. Final products were formulated at 1 mg/mL concentration in RNase-free 1 mM sodium citrate buffer, pH 6.4.

### Lipid Nanoparticle Formulation

Lipid nanoparticles were prepared through a modification of the ethanol injection technique. The initial step involved dissolving the component lipids (lipidoids, helper lipids, cholesterol, and C14-PEG2000) in ethanol, with each component at 1–10 mg/mL concentration. These lipid components were combined at specific molar proportions: lipidoids (35), helper lipids (40), cholesterol (22.5), and PEG-lipid (2.5). This lipid solution was then combined with mRNA that had been prepared in 10 mM sodium citrate buffer (pH 3.0), maintaining a lipidoid to mRNA weight ratio of 10:1. Following particle formation, residual ethanol was removed by dialyzing the LNP suspension against PBS using Thermo Scientific cassettes with a 3 kDa molecular weight cutoff.

### Lipid Nanoparticle Characterization

Lipid nanoparticles were diluted to a concentration of 0.0005 mg/mL with PBS. Size distribution, polydispersity index (PDI), and zeta potential of formulated Lipid nanoparticles were measured using Synergy H1 plate reader (BioTek). RNA entrapment efficiency was measured using the Quant-iT RiboGreen RNA Assay Kit (ThermoFisher) and a Synergy H1 plate reader (BioTek) according to manufacturer instructions. Ionization potential (surface p*K*_a_) of the LNP was measured at a dilution of 0.05 mg/mL using the 2-(*p*-toluidinyl) naphthalene-6-sulfonic acid (TNS) assay as previously described.^35^ Briefly, LNPs were added to buffers of varying pHs, and the fluorescence of TNS which exhibits signal in the presence of positively charged membranes was measured with a Synergy H1 plate reader (BioTek).

### AAV Vectors

Cas9 plasmid was purchased from Addgene (AAV-CMVc-Cas9 #106431). The mCherry construct was a premade AAV vector purchased from PackGene Biotech, (ssAAV-CAG-mCherry.WPRE.SV40pA, cat. no. AAV-EA022).

### AAV Packaging and Purification

AAV plasmid integrity was verified through *Sma*I restriction enzyme digestion, specifically examining ITR regions. Following validation, these constructs served as templates for AAV2-Cas9 and AAV2-mCherry viral production by PackGene Biotech, LLC. Viral titers were determined using Real-time SYBR Green PCR against standard curves prepared from linearized parental AAV vectors, establishing concentrations of 1.5 × 10^13^ GC/ml.

### Cell Culture

Neuro-2a cells (purchased from ATCC) were cultured at 37 °C in a 5% CO_2_ environment. The culture medium consisted of Dulbecco’s modified Eagle’s medium (DMEM) purchased from Life Technologies supplemented with the following components: FBS (10%, Life Technologies), sodium pyruvate (1.0 mM, Life Technologies), glutamine (2 mM), and a streptomycin-penicillin antibiotic mixture (1%, Gibco).

### Transfection of *In Vitro* Cultured Cells

For *in vitro* transfection studies, Neuro-2a cells we seeded in 24-well plates at a density of approximately 50,000 cells per well. The following day, transfection was using Lipofectamine LTX to deliver multiple plasmid components: Cas9 nuclease (50 ng), gRNA (10–100 ng), ZFR (150 ng), dCas9-HP1a-KRAB (100 ng), YFP for monitoring transfection efficiency (25 ng), and a puromycin resistance marker (50 ng). Twenty-4 h following transfection, puromycin selection was done using 0.5 μg/mL concentration (Gibco-life tech)

### Quantitative RT-PCR (qRT-PCR) Analysis

For RNA extraction, cell lysis was performed, and RNA was extracted using either Life Technologies’ Trizol or Qiagen’s RNAEasy Plus Mini Kit. The resulting RNA underwent reverse transcription to cDNA using Thermo Fisher’s High-Capacity RNA-to-cDNA Kit. Quantitative PCR analysis employed SYBR Green PCR Master Mix (Thermo Fisher), with 18S rRNA serving as the normalization standard. We calculated relative expression changes using the 2^–ΔΔCt^ method, comparing against control group values. The complete list of primers used for quantitative PCR are in Table S4.

### Plasma Biomarker Analysis of Liver Function

The isolation of plasma from blood samples was achieved by centrifugation, performed for 10 min at 2,000 × g while maintaining 4 °C temperature. Plasma bile acids and alanine aminotransferase (ALT) levels were analyzed by IDEXX Laboratories (IDEXX Reference Laboratories, USA). Analysis was performed using the IDEXX Catalyst chemistry analyzer system. Total bile acids were measured through an enzymatic cycling reaction utilizing 3α-hydroxysteroid dehydrogenase (reported in μmol/L). ALT activity was determined via a coupled enzymatic assay monitoring the rate of NADH oxidation spectrophotometrically (reported in IU/L). All assays were performed with appropriate calibration controls following manufacturer’s validated protocols.

### ELISA-Based Chemiluminescent Assay for Plasma Cytokine Analysis

Multiplexed analysis of plasma cytokines was conducted at the UPMC Cancer Proteomics Facility: Luminex Core Laboratory using two magnetic bead-based immunoassay panels. A Mouse High Sensitivity T Cell 8-plex kit (MHSTCMAG-70K-08, Millipore) was used to measure IL-6, IL-4, IL-2, IL-1β, IL-1α, IL-17A, IL-10, and IFN-γ from 60 μL plasma samples. MIP-1α levels were determined using the Mouse Cytokine MAGNETIC Panel 1 (MCYTOMAG-70K-01) from 35 μL plasma samples. All samples were analyzed in duplicate following manufacturer’s protocols. Briefly, samples and calibrators were incubated with analyte-specific antibody-conjugated magnetic beads in 96-well plates. After washing, biotinylated detection antibodies were added followed by streptavidin–horseradish peroxidase. Bead fluorescence was measured using a Luminex analyzer, and cytokine concentrations were calculated against standard curves using xPONENT software.

### Anti-AAV2 Antibody Assays

Anti-AAV2 antibodies were quantified utilizing both *in vitro* neutralization and ELISA assays at the Gene Therapy Program’s Immunology Core facility at the University of Pennsylvania’s Perelman School of Medicine (Philadelphia, PA).

### IgG and IgG2B ELISA

The immunoassay utilized Nunc maxisorp plates (Thermo Fisher Scientific, Waltham, MA) with an initial AAV2 particle coating step (2 × 10^10^ particles/mL in carbonate buffer, pH 9.6) performed overnight at 4 °C. Standard curves were generated using Sigma-Aldrich (St. Louis, MO) purified immunoglobulins (IgG and IgG2B) in serial dilutions. Following a room temperature blocking step (1 h) with PBS containing 2% BSA and 0.05% Tween-20, we applied diluted plasma samples in duplicate wells for a 3 h room temperature incubation. Detection employed two horseradish peroxidase (HRP)-conjugated secondary antibodies from Southern Biotech: anti-mouse IgG-HRP (1:20,000) and anti-mouse IgG2B-HRP (1:10,000). After incubating at 37 °C for 1 h and washing, we developed the plates using SIGMAFASTTM OPD substrate (Sigma-Aldrich) according to manufacturer specifications, with absorbance readings taken at 492 nm.

### Neutralizing Antibody Assay

Anti-AAV2 neutralizing antibody levels were evaluated in selected plasma specimens using a cell-based *in vitro* approach. The protocol starts with seeding HEK293 cells (1 × 10^5^ cells/well) into 96-well plates, allowing 24 h for attachment. Test samples were then prepared by heat inactivation followed by serial dilution, combining them with luciferase-expressing AAV2 vector (1 × 10^4^ viral particles/cell) for a 1 h incubation at 37 °C. This mixture was introduced to the cells for a 24 h incubation period. Using the Galacto-Star System (Applied Biosystems), luciferase activity was measured. The neutralizing antibody titer was defined as the maximum sample dilution that achieved at least 50% reduction in luciferase expression compared to controls without inhibition. For reference, a 1:10 neutralizing antibody titer indicates that a sample diluted 10-fold exhibits a luciferase signal below 50% of the noninhibition control value.

### Animals

Our animal research protocols adhered to established laboratory animal care and usage guidelines, with full approval from the University of Pittsburgh’s Institutional Animal Care and Use Committee (IACUC). All experiments followed institutional protocols and included both male and female C57BL/6 mice (purchased from JAX, Stock #000664) aged 6–8 weeks. Each experimental group contained a minimum of three animals, with precise group sizes documented in the corresponding figure legends. C57BL/6 mouse strain was utilized for both AAV LPS and experiments.

### Retro-orbital Injections

For administration of AAV particles, the retro-orbital route targeting the venous sinus was selected. Mice were anesthetized using isoflurane at 3% concentration, after which 100 μL of AAV solution, containing between 1 × 10^11^ and 1 × 10^12^ genome copies per animal, was injected into each mouse’s left eye.

### Tissue Harvest

Using CO_2_ inhalation for euthanasia, tissue samples were collected from multiple organs—liver, spleen, lung, and bone marrow—as well as blood samples. Each tissue sample was immediately placed in RLT Plus buffer from Qiagen, followed by preservation through snap freezing methods for later RNA extraction and analysis.

### *In Vivo* LPS Administration

*Escherichia coli* strain 0127:B8-derived lipopolysaccharides were administered via intraperitoneal (i.p.) route (LPS purchased from Sigma-Aldrich, St. Louis, MO, USA). The LPS was prepared as a 2.5 mg/mL solution in PBS. At 26 h following LPS administration (as depicted in the experimental timeline schematics), animals were euthanized using CO_2_ inhalation.

### Statistical Analysis and Reproducibility

All *in vitro* experiments were conducted in triplicate, with consistency observed across replicates. For animal studies, a minimum of three biological replicates were performed, yielding reproducible results. The assignment of mice to experimental or control groups was performed randomly, though experimenters were not blinded during data acquisition or analysis. Data are presented as the mean ± SEM. The variable *N* denotes either the number of individual transfections (for *in vitro* work) or the number of animals (for *in vivo* studies). Statistical analyses were performed using GraphPad’s Prism 10 Software, which are detailed within figure legends. When comparing two groups for statistical significance, two-tailed unpaired *t* tests was employed, while multiple group analyses was performed by one-way ANOVA with Dunnett’s multiple comparisons test. *P ≤ 0.05 was considered significant (with additional thresholds at **P ≤ 0.01, ***P ≤ 0.001, ****P ≤ 0.0001).

### Sample Size Determination and Exclusion Criteria

Initial sample sizes for all experiments were determined based on power analysis to detect a 50% change in primary outcomes with 80% power at α = 0.05. Exclusion criteria were pre-established as follows: (1) Plasma cytokine analysis: samples with cytokine levels outside the detection limit of the assay were excluded. (2) Flow cytometry: samples with cell viability below 70% were excluded from analysis. (3) Animal studies: animals showing signs of injection failure (subcutaneous leakage, incomplete injection volume) were excluded from the study immediately after injection.

## Supplementary Material



## Data Availability

The raw and processed data supporting the findings of this study are available from the corresponding author subject to reasonable request. All materials are available upon completion of a material transfer agreement.

## Notes

*In vitro* and *in vivo* evaluation of *Myd88*-targeting zinc finger and CRISPR-dCas9 mRNA delivered by 306O_10_ lipid nanoparticles, including repressor screening, LNP physicochemical characterization, biodistribution, immune cell targeting, and safety assessment following repeated dosing (Figures S1–S8) and zinc finger target sequences and genomic positions, lipid nanoparticle characterization parameters, zinc finger DNA sequences, and mouse qPCR primer sequences (Tables S1–S4) (PDF)
