# Supplementary material for: Enhanced Epigenetic Modulation via mRNA-Encapsulated Lipid Nanoparticles Enables Targeted Anti-inflammatory Control
Source: ACS Synth Biol. 2026 Jan 15;15(2):400–10. doi: 10.1021/acssynbio.5c00188 (PMC12930510; doi:10.1021/acssynbio.5c00188)
Supplement: Supplementary file 1 [file sb5c00188_si_001.pdf]

## Supporting Information

# Enhanced epigenetic modulation via mRNA-encapsulated lipid nanoparticles enables targeted anti-inflammatory control

Tahere Mokhtari[1-3], Mohammad N. Taheri[1-3], Sarah Akhlaghi[1,2], Armin Aryannejad[1,2], Yuda Xiang[1,6], Vineet Mahajan[1,2], Kamyar Keshavarz[1-3], Amirreza Kiani[4,7], Samantha Yang[5], Samuel LoPresti[5], Ryan LeGraw[1,2], Kathryn A. Whitehead[5], Samira Kiani[1-3,7,8] *

Affiliations:

1. Division of Experimental Pathology, Department of Pathology, University of Pittsburgh School of Medicine, Pittsburgh, PA, USA
2. Pittsburgh Liver Research Center, University of Pittsburgh School of Medicine, Pittsburgh, PA, USA
3. Department of Bioengineering, University of Pittsburgh, Pittsburgh, PA, USA
4. Department of Electrical and Computer Engineering, University of Toronto, Toronto, ON, CA
5. Department of Chemical Engineering, Carnegie Mellon University, Pittsburgh, PA, USA
6. School of Medicine, Tsinghua Medicine, Tsinghua University, Beijing, China
7. GenexGen Inc., 169 E Portola Ave, Los Altos, CA
8. HeXembio Inc. 877 Francisco Street, Los Angeles, CA

*Correspondence: samira.kiani@pitt.edu

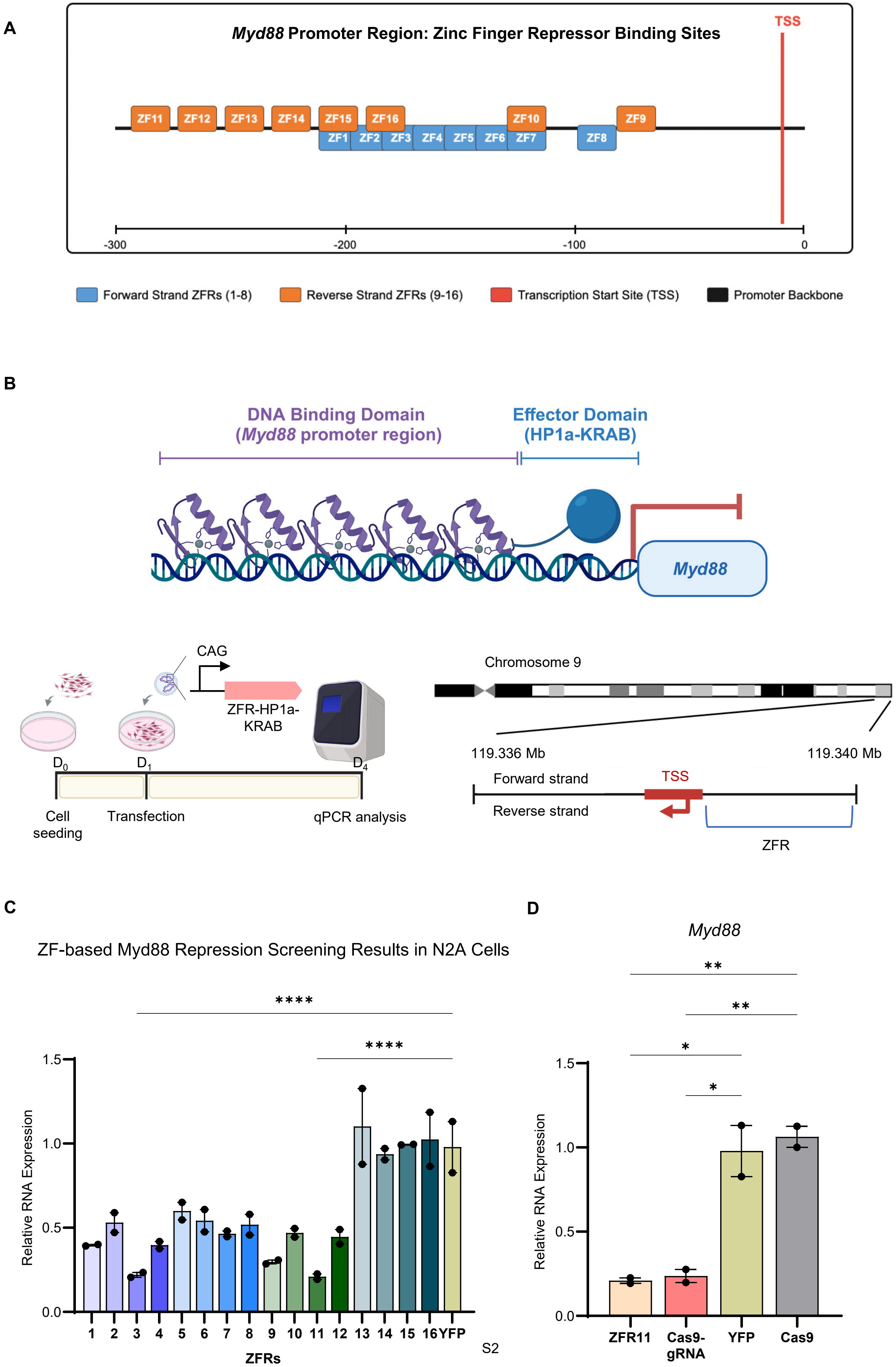
A
Myd88 Promoter Region: Zinc Finger Repressor Binding Sites
TSS
ZF11
ZF12
ZF13
ZF14
ZF15
ZF16
ZF10
ZF9
ZF1
ZF2
ZF3
ZF4
ZF5
ZF6
ZF7
ZF8
-300
-200
-100
0
Forward Strand ZFRs (1-8)
Reverse Strand ZFRs (9-16)
Transcription Start Site (TSS)
Promoter Backbone
B
DNA Binding Domain
(Myd88 promoter region)
Effector Domain
(HP1a-KRAB)
Myd88
CAG
ZFR-HP1a-KRAB
D0
D1
D4
Cell seeding
Transfection
qPCR analysis
Chromosome 9
119.336 Mb
119.340 Mb
Forward strand
Reverse strand
TSS
ZFR
C
ZF-based Myd88 Repression Screening Results in N2A Cells
****
****
Relative RNA Expression
1.5
1.0
0.5
0.0
1
2
3
4
5
6
7
8
9
10
11
12
13
14
15
16
YFP
ZFRs
D
Myd88
**
**
*
*
ZFR11
Cas9-gRNA
YFP
Cas9
S2

**Figure S1.** Evaluating zinc-finger HP1a-Krab *Myd88* repression *in vitro.* (A) Schematic representation of 16 ZFRs targeting the mouse *Myd88* promoter across a 300 bp region upstream of the transcription start site (TSS, red line). Forward strand-targeting ZFRs (ZF1-ZF8, blue boxes) and reverse strand-targeting ZFRs (ZF9-ZF16, orange boxes) are positioned above and below the promoter backbone (black line), respectively. Scale bar indicates distance in base pairs upstream from the TSS. (B) Top: Schematic of ZF-based artificial transcription factor (ATF) recruitment to the *Myd88* promoter region. ZF-based ATF is composed of an effector domain and a DNA-binding domain. Bottom left: Schematic of the experimental design. Mouse neuroblastoma (N2a) cells were transfected with either one of 16 different *Myd88*-targeting ZF-based repressors or yellow fluorescent protein (YFP)-expressing plasmid as a control. *Myd88* mRNA expression levels were analyzed by qRT-PCR three days post-transfection. Bottom right: Schematic representation of the ZFR11 binding sites within the Myd88 promoter region. (C) Fold changes in Myd88 mRNA transcript levels were quantified relative to the YFP control group (n = 2 biologically independent samples). Data are presented as mean + s.e.m. Statistical analysis was performed using one-way analysis of variance (ANOVA) followed by Dunnett's multiple comparisons test. Differences were considered statistically significant at $^*P \leq 0.05$. ZFR1 through ZFR12 showed significant differences compared to the YFP control, while ZFR13 through ZFR16 showed no significant differences. For clarity of data presentation, statistical significance is shown only for ZFR3 and ZFR11 with **** $P \leq 0.0001$). (D) Fold changes in *Myd88* mRNA transcript levels were quantified relative to the YFP group ($N = 2$ biologically independent samples). Data are presented as mean + s.e.m. Statistical analysis was performed using one-way analysis of variance (ANOVA) followed by Dunnett's multiple comparisons test. Differences were considered statistically significant at $^*P \leq 0.05$.

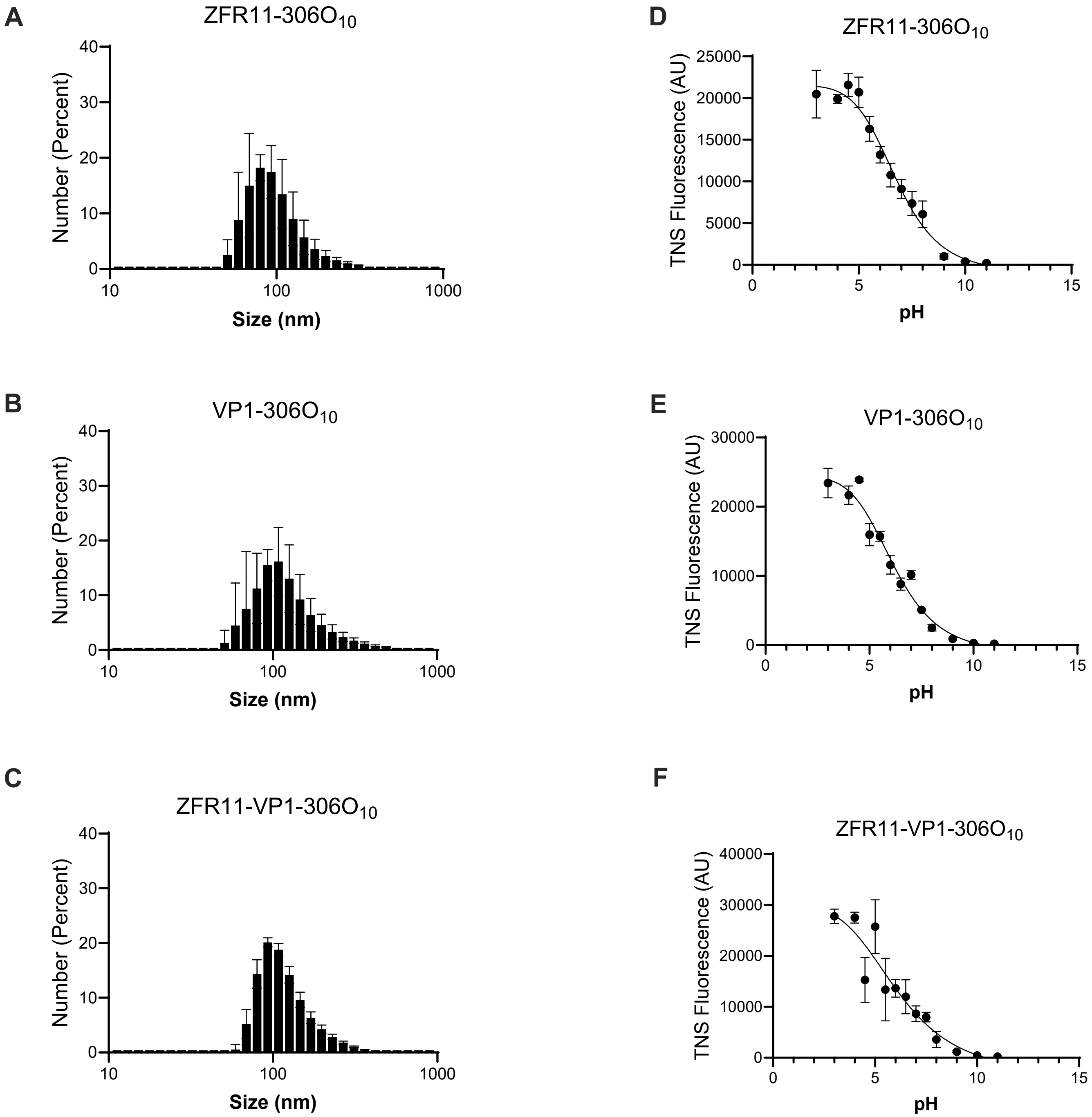
A
ZFR11-306O10
Number (Percent)
Size (nm)
B
VP1-306O10
Number (Percent)
Size (nm)
C
ZFR11-VP1-306O10
Number (Percent)
Size (nm)
D
ZFR11-306O10
TNS Fluorescence (AU)
pH
E
VP1-306O10
TNS Fluorescence (AU)
pH
F
ZFR11-VP1-306O10
TNS Fluorescence (AU)
pH

**Figure S2.** Characterization of $306O_{10}$ lipid nanoparticles. (A-C) Size distribution measured by dynamic light scattering for ZFR11- $306O_{10}$ (N=3) , VP1- $306O_{10}$ (N=3), and ZFR11-VP1- $306O_{10}$ (N=3) LNPs. (D-F) pH-dependent ionization profiles determined by TNS assay showing pKa values of 6.76, 6.16, and 6.08 for scattering for ZFR11- $306O_{10}$ (N=3) , VP1- $306O_{10}$ (N=3), and ZFR11-VP1- $306O_{10}$ (N=3) LNPs, respectively. Data are presented as mean ± SD.

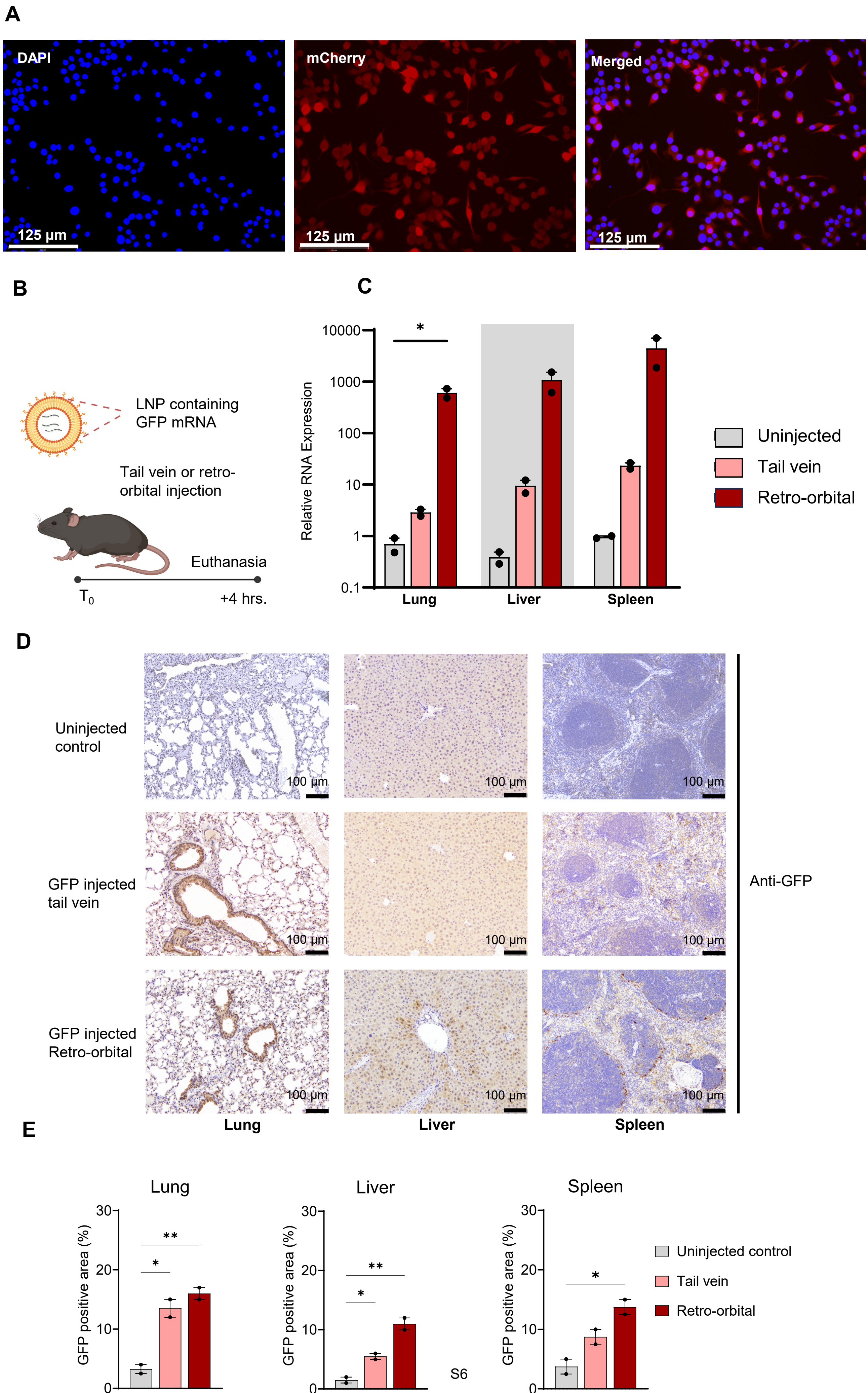
A
DAPI
125 µm
mCherry
125 µm
Merged
125 µm
B
LNP containing
GFP mRNA
Tail vein or retro-
orbital injection
Euthanasia
$T_0$
+4 hrs.
C
Relative RNA Expression
10000
1000
100
10
1
0.1
*
Lung
Liver
Spleen
Uninjected
Tail vein
Retro-orbital
D
Uninjected
control
GFP injected
tail vein
GFP injected
Retro-orbital
100 µm
Anti-GFP
Lung
Liver
Spleen
E
Lung
Liver
Spleen
GFP positive area (%)
30
20
10
0
**
*
Uninjected control
Tail vein
Retro-orbital

S6

**Figure S3.** Evaluating $306O_{10}$ transfection efficiency and biodistribution. (A) Fluorescence microscopy images of RAW 264.7 culture 24 hours post-transfection. Images show DAPI (nuclear staining), mCherry (anti-mCherry immunofluorescence staining), and merged channels. Scale bar, 125 μm. (B) Schematic representation of GFP-$306O_{10}$ lipid nanoparticle delivery to C57BL/6 mice (1.2 mg mRNA/kg mouse). (C) qRT-PCR analysis of GFP expression levels in lung, liver, and spleen tissues from C57BL/6 mice four hours after systemic injection of GFP-$306O_{10}$ via either retro-orbital (RO) or tail vein (TV) routes (N = 2 mice per group) or no injection (control, N = 2 mice). Data are normalized to uninjected controls and presented as mean + s.e.m. Statistical analysis was performed using one-way analysis of variance (ANOVA) followed by Dunnett's multiple comparisons test. $^{*}P \leq 0.05$ was considered statistically significant. (D) Representative immunohistochemical staining for GFP in lung, liver, and spleen tissue sections from mice receiving tail vein injection (N = 2), retro-orbital injection (N = 2), or no injection (control, N = 2). Scale bar, 100 μm. E) Quantification of GFP staining intensity (Uninjected control: N = 2, Tail vein GFP injected: N = 2, Retro-orbital GFP injected: N =2). Data are presented as mean + s.e.m. Statistical analysis was performed using one-way ANOVA followed by Dunnett's multiple comparisons test. $^{*}P \leq 0.05$ was considered statistically significant.

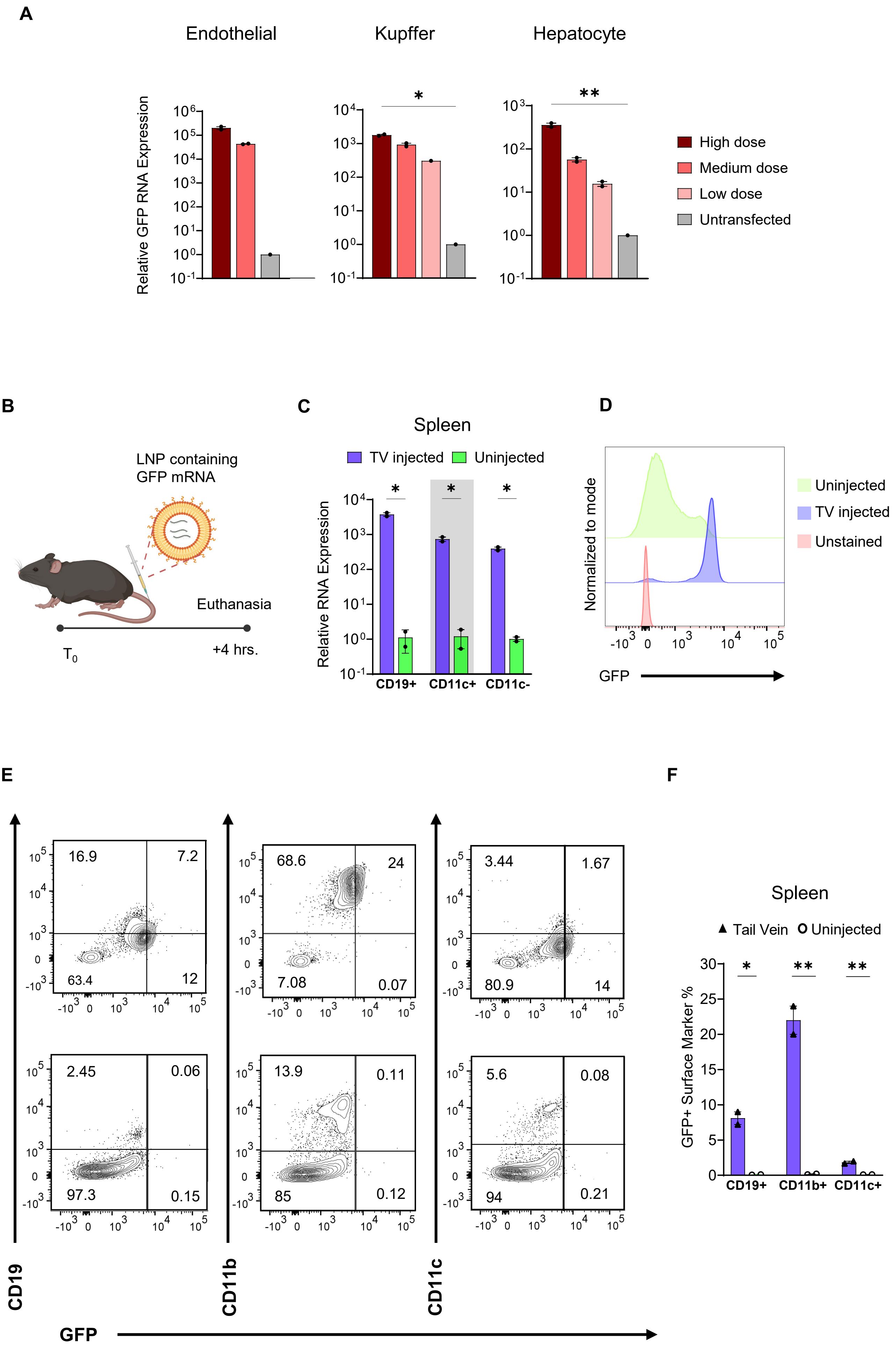
A
Endothelial
Kupffer
Hepatocyte
Relative GFP RNA Expression
High dose
Medium dose
Low dose
Untransfected
B
LNP containing
GFP mRNA
Euthanasia
$T_0$
+4 hrs.
C
Spleen
TV injected
Uninjected
Relative RNA Expression
CD19+
CD11c+
CD11c-
D
Normalized to mode
GFP
Uninjected
TV injected
Unstained
E
16.9
7.2
63.4
12
2.45
0.06
97.3
0.15
68.6
24
7.08
0.07
13.9
0.11
85
0.12
3.44
1.67
80.9
14
5.6
0.08
94
0.21
CD19
CD11b
CD11c
GFP
F
Spleen
Tail Vein
Uninjected
GFP+ Surface Marker %
CD19+
CD11b+
CD11c+

**Figure S4.** Cell type-specific and immune population targeting analysis of $306O_{10}$ lipid nanoparticles. (A) GFP mRNA expression in primary liver cell populations (LSECs, Kupffer cells, and hepatocytes) 48 hours after GFP-$306O_{10}$ transfection at three concentrations (high: 0.01 mg/mL, medium: 0.005 mg/mL, low: 0.0025 mg/mL). Fold changes were quantified relative to untransfected controls (N = 2 biologically independent samples). Data are presented as mean + s.e.m. Statistical analysis was performed using one-way ANOVA followed by Dunnett's multiple comparisons test. *$P \leq 0.05$ was considered statistically significant. (B) Schematic representation of experimental design for characterizing $306O_{10}$ immune cell targeting in spleen. C57BL/6J mice received GFP-$306O_{10}$ (1.5 mg $kg^{-1}$) via tail vein injection. (C) GFP mRNA expression in CD19+, CD11c+, and CD11c- populations isolated by magnetic-activated cell sorting from spleen tissues 4 hours post-injection (N = 2 mice per group). Data are presented as mean + s.e.m. Statistical analysis was performed using the unpaired parametric t-test. *$P \leq 0.05$ was considered significant. (D) Representative flow cytometry histograms demonstrating a rightward shift in GFP fluorescence signal in spleen samples from tail vein injected mice compared to both unstained and uninjected controls (N = 2 mice per group). (E-F) Quantitative flow cytometry analysis showing percentage of GFP-positive cells within CD19+ (7.2%), CD11b+ (24%), and CD11c+ (2%) populations in spleen tissue following GFP-$306O_{10}$ administration, compared to matching uninjected controls. Data are presented as mean + s.e.m. Statistical analysis was performed using unpaired parametric t-test. *$P \leq 0.05$ was considered statistically significant.

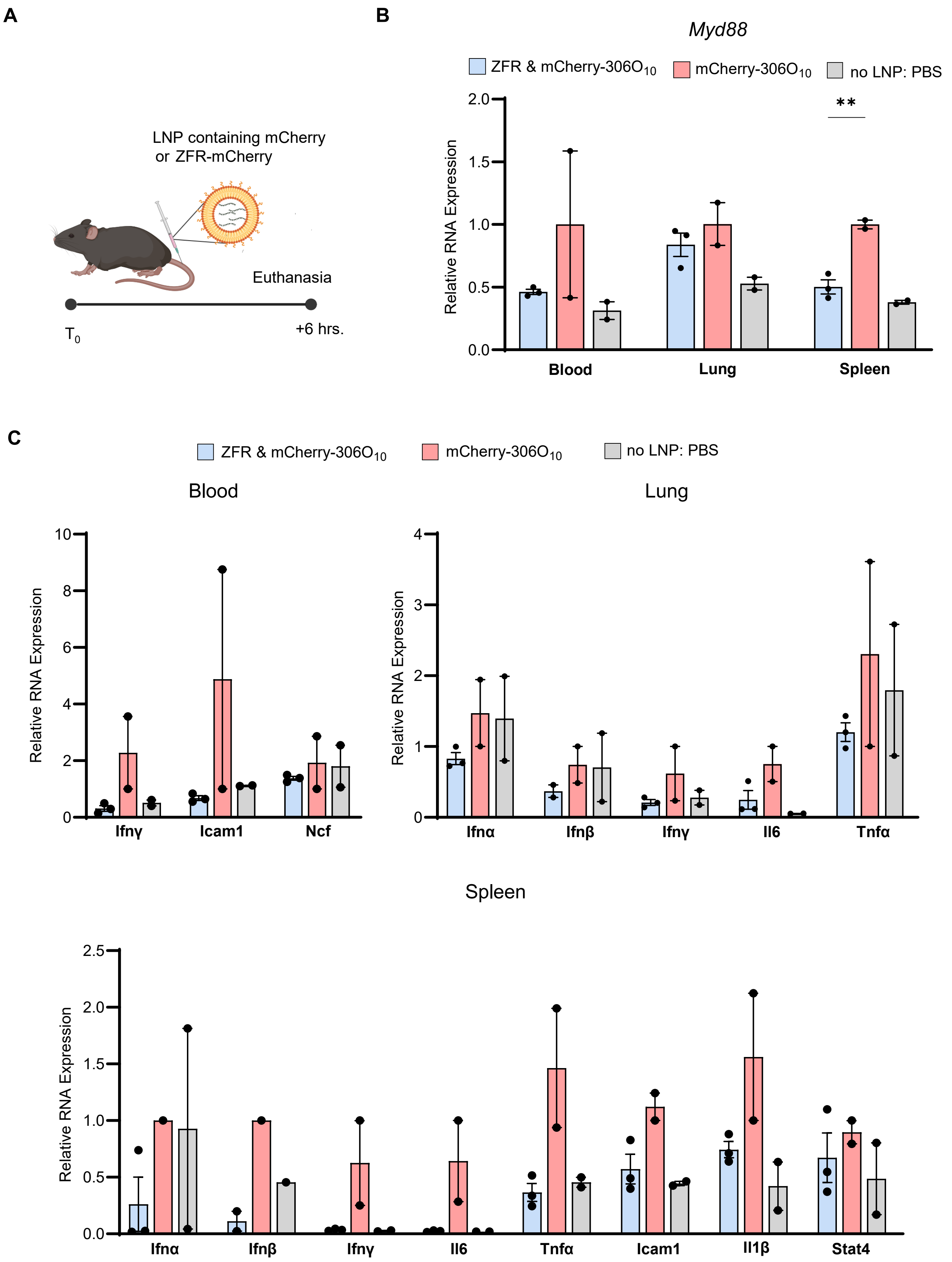
A
LNP containing mCherry
or ZFR-mCherry
Euthanasia
T0
+6 hrs.
B
Myd88
ZFR & mCherry-306O10
mCherry-306O10
no LNP: PBS
**
Relative RNA Expression
2.0
1.5
1.0
0.5
0.0
Blood
Lung
Spleen
C
ZFR & mCherry-306O10
mCherry-306O10
no LNP: PBS
Blood
Relative RNA Expression
10
8
6
4
2
0
Ifnγ
Icam1
Ncf
Lung
Relative RNA Expression
4
3
2
1
0
Ifnα
Ifnβ
Ifnγ
Il6
Tnfα
Spleen
Relative RNA Expression
2.5
2.0
1.5
1.0
0.5
0.0
Ifnα
Ifnβ
Ifnγ
Il6
Tnfα
Icam1
Il1β
Stat4

**Figure S5.** Delivery of lipid nanoparticles carrying mRNA encoding *Myd88*-targeting zinc finger enables immunomodulation under homeostatic conditions. (A) Schematic representation of experimental design. C57BL/6 mice received intravenous injection of either mCherry-306$O_{10}$ or ZFR11-mCherry-306$O_{10}$ (0.5 mg $kg^{-1}$). Tissues were collected six hours post-administration for analysis. (B) qRT-PCR analysis of *Myd88* expression in blood, lung, and spleen tissues six hours after zinc finger-mediated repression (N = 3 mice for ZFR11-mCherry-306$O_{10}$ group, N = 2 for mCherry-306$O_{10}$ control group). Data are presented as mean + s.e.m. Statistical analysis was performed using the unpaired parametric t-test. *$P \leq 0.05$ was considered statistically significant. (C) qRT-PCR analysis of inflammatory gene expression (*Ifn-α*, *Ifn-β*, *Ifn-γ*, *Icam-1*, *Ncf*, *Il6*, *Tnf-a*, *Il-1β*, and *Stat4*) across different tissues. Expression levels were normalized to matched tissue samples from mCherry-306$O_{10}$-treated control mice (N = 6 mice for ZFR11-mCherry-306$O_{10}$ group, N = 3 for mCherry-306$O_{10}$ group). Data are presented as mean + s.e.m. Statistical analysis was performed using the unpaired parametric t-test. *$P \leq 0.05$ was considered statistically significant.

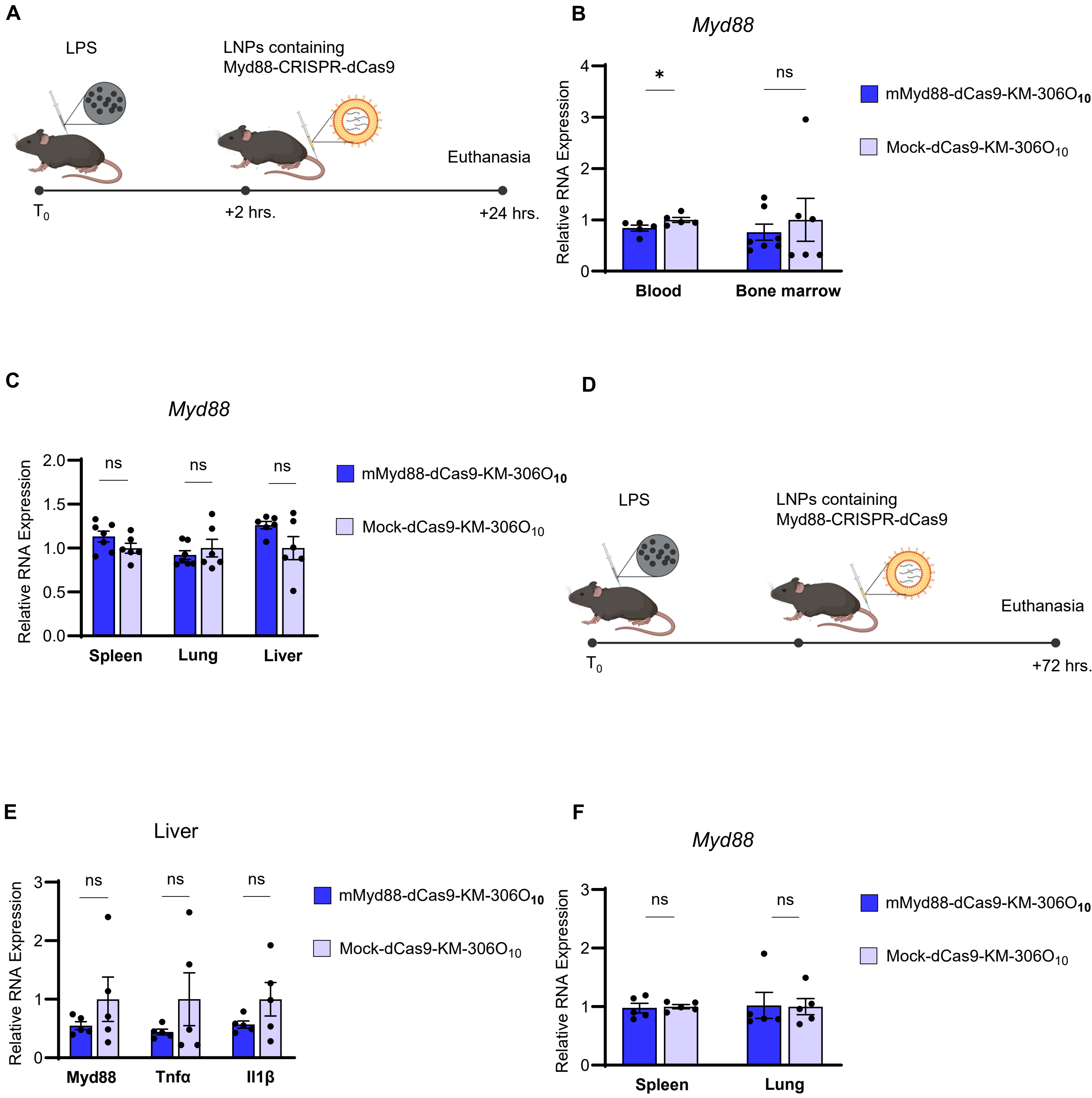
A
LPS
LNPs containing
Myd88-CRISPR-dCas9
Euthanasia
T0
+2 hrs.
+24 hrs.
B
Myd88
Relative RNA Expression
0
1
2
3
4
*
ns
Blood
Bone marrow
mMyd88-dCas9-KM-306O10
Mock-dCas9-KM-306O10
C
Myd88
Relative RNA Expression
0.0
0.5
1.0
1.5
2.0
ns
ns
ns
Spleen
Lung
Liver
mMyd88-dCas9-KM-306O10
Mock-dCas9-KM-306O10
D
LPS
LNPs containing
Myd88-CRISPR-dCas9
Euthanasia
T0
+72 hrs.
E
Liver
Relative RNA Expression
0
1
2
3
ns
ns
ns
Myd88
Tnfα
Il1β
mMyd88-dCas9-KM-306O10
Mock-dCas9-KM-306O10
F
Myd88
Relative RNA Expression
0
1
2
3
ns
ns
Spleen
Lung
mMyd88-dCas9-KM-306O10
Mock-dCas9-KM-306O10

**Figure S6.** Delivery of lipid nanoparticles carrying mRNA encoding *Myd88*-targeting CRISPR-dCas9 shows limited efficacy in *Myd88* repression during LPS-induced inflammation. (A) Schematic representation of experimental design. C57BL/6 mice received intraperitoneal injection of LPS (2.5 mg $kg^{-1}$) followed by tail vein administration of $306O_{10}$ LNPs encapsulating dCas9-KRAB-MeCP2 mRNA with either *Myd88*-targeting gRNA or non-targeting mock gRNA controls (dCas9-KRAB-MeCP2 1.6 mg $kg^{-1}$ and gRNA 0.4 mg $kg^{-1}$) 2 hours later. Tissues were collected 24 hours post-LNP administration. (B) qRT-PCR analysis of *Myd88* expression in blood and bone marrow tissues following LPS challenge and CRISPR-mediated repression (N = 5 mice for dCas9-KRAB-MeCP2-*Myd88* gRNA group in blood, N = 5 for mock gRNA control group in blood, N = 7 mice for dCas9-KRAB-MeCP2-*Myd88* gRNA group in bone marrow, N = 6 for mock gRNA control group in bone marrow). Data are presented as mean + s.e.m. (C) qRT-PCR analysis of *Myd88* mRNA expression in spleen, liver, and lung. Expression levels were normalized to mock gRNA-treated control mice. Data are presented as mean + s.e.m. (D) Schematic representation of extended experimental design. C57BL/6 mice received intraperitoneal injection of LPS (2.5 mg $kg^{-1}$) followed by tail vein administration of dCas9-KRAB-MeCP2-$306O_{10}$ constructs 2 hours later. Tissues were collected 72 hours post-LNP administration. (E) qRT-PCR analysis of *Myd88*, *Il1b*, and *Tnfa* expression levels in liver tissue 72 hours following LPS injection and CRISPR-mediated therapy (N = 5 mice for dCas9-KRAB-MeCP2-*Myd88* gRNA group, N = 5 for mock gRNA control group). Data are presented as mean + s.e.m. (F) qRT-PCR analysis of *Myd88* mRNA expression in spleen and lung tissues. Expression levels were normalized to mock gRNA-treated control mice (N = 5 mice for dCas9-KRAB-MeCP2-*Myd88* gRNA group, N = 5 for mock gRNA control group). Data are presented as mean + s.e.m. Statistical analysis was performed using the unpaired parametric t-test. $^*P \leq 0.05$ was considered statistically significant.

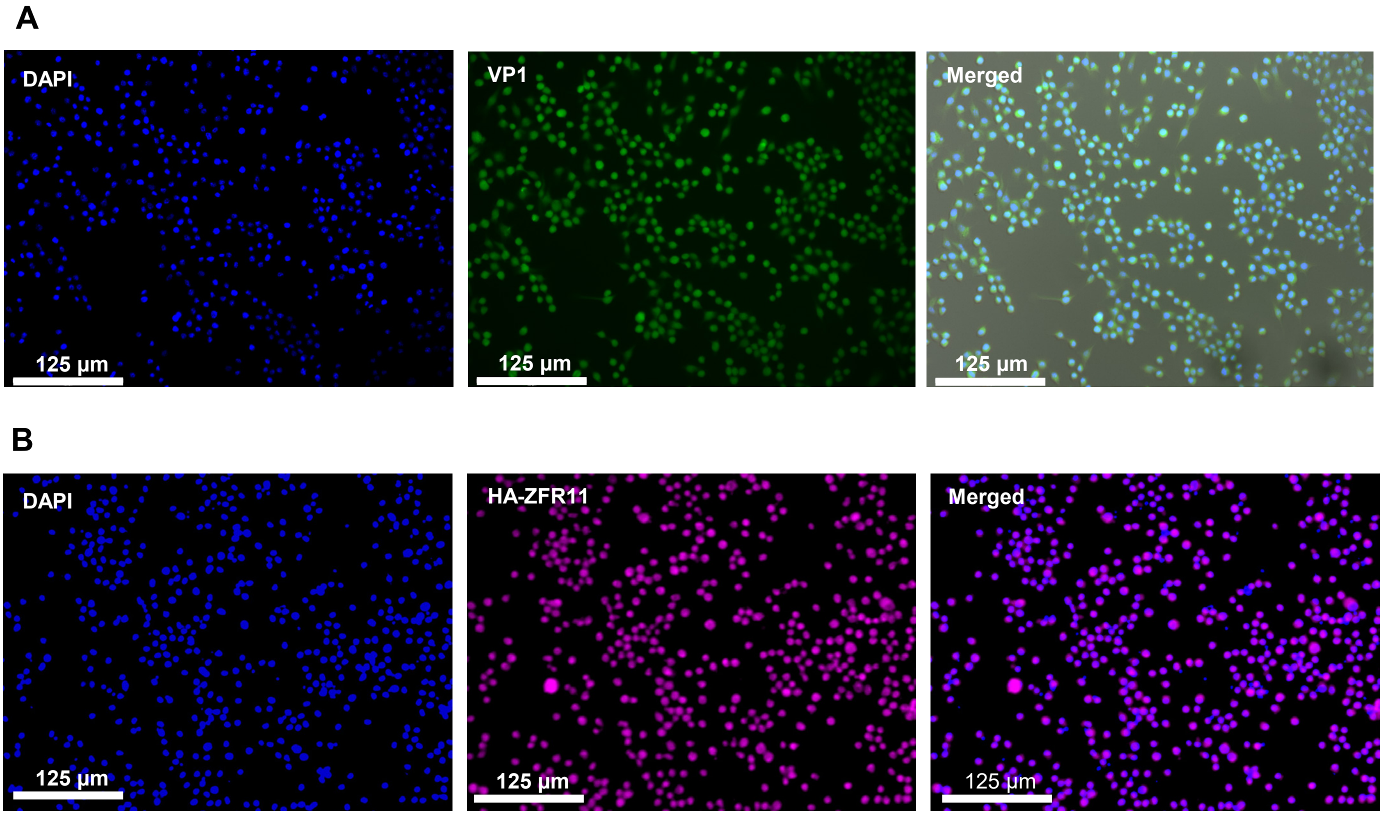
A
DAPI
125 µm
VP1
125 µm
Merged
125 µm
B
DAPI
125 µm
HA-ZFR11
125 µm
Merged
125 µm

**Figure S7.** In vitro validation of VP1 and ZFR11 protein expression from $306O_{10}$ LNP-delivered mRNA. (A) Immunofluorescence microscopy confirms successful VP1 protein expression in RAW 264.7 macrophages 24 hours post-transfection with VP1 mRNA-loaded $306O_{10}$ LNPs (0.01 mg/mL, N=3). Cells were fixed and stained with anti-VP1 antibody (green) and DAPI nuclear stain (blue). Images show DAPI (nuclear staining), VP1 (anti-VP1 immunofluorescence staining), and merged channels. (B) Confirmation of ZF11-HP1a-KRAB protein expression in RAW 264.7 macrophages 24 hours post-transfection with HA-tagged ZFR11 mRNA-loaded $306O_{10}$ LNPs (0.01 mg/mL, N=3). Images show DAPI (nuclear staining), ZFR11 (anti-HA HA-tagged ZFR11 immunofluorescence staining), and merged channels. Scale bars: 125 µm. These results validate the functional delivery and translation of both components of our anti-adjuvant vaccine platform prior to in vivo experimentation.

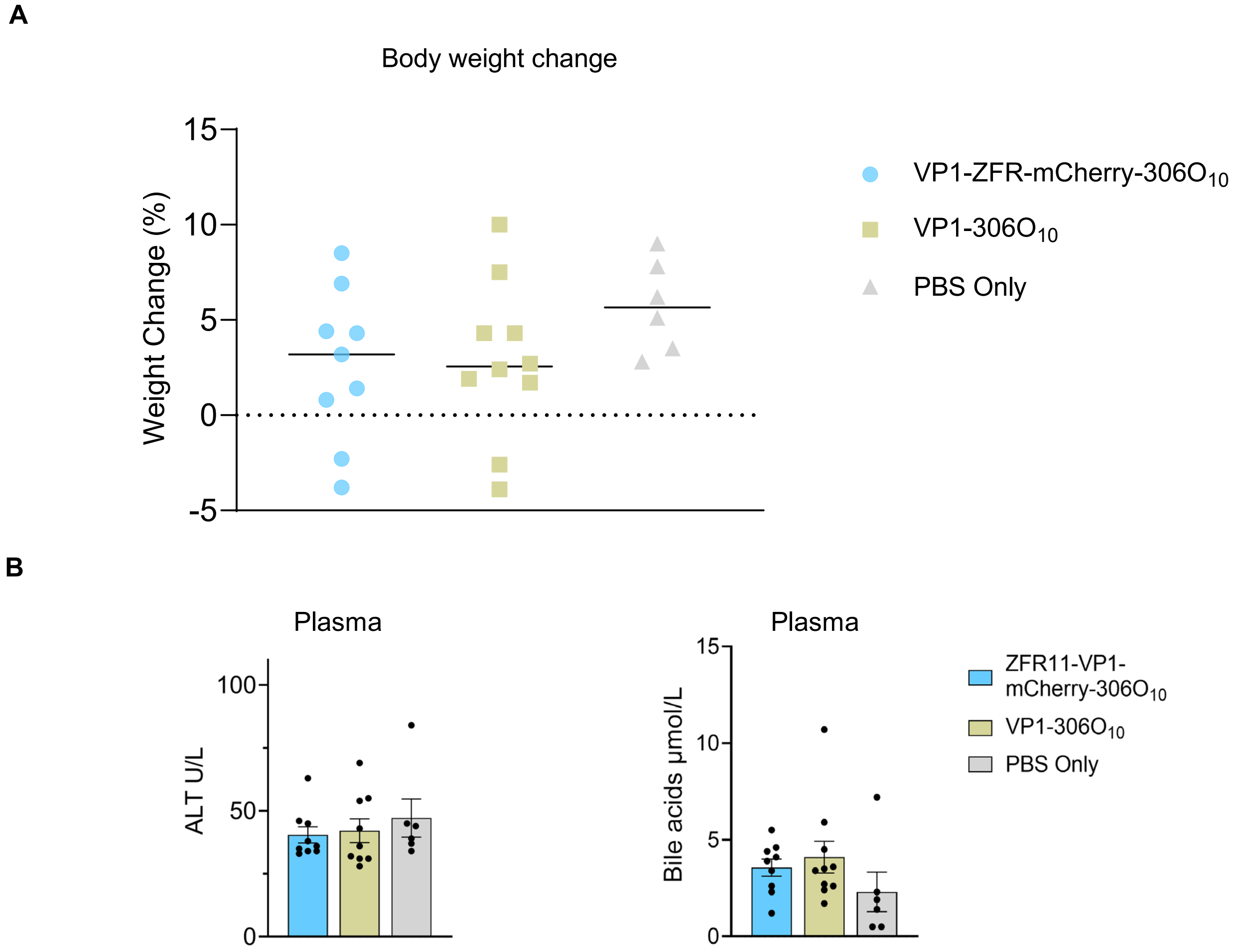
A
Body weight change
Weight Change (%)
15
10
5
0
-5
VP1-ZFR-mCherry-306O10
VP1-306O10
PBS Only
B
Plasma
ALT U/L
100
50
0
Plasma
Bile acids µmol/L
15
10
5
0
ZFR11-VP1-mCherry-306O10
VP1-306O10
PBS Only

**Figure S8.** Safety assessment of repeated LNP-mediated therapeutic mRNA delivery in AAV pre-immunized mice. **(A)** Percent body weight change from baseline at day 0 (before first LNP injection) to endpoint at day 56 (harvest) in C57BL/6J mice receiving weekly LNP injections as part of the AAV pre-immunization study. Mice received four weekly intravenous injections of LNP-encapsulated mRNA encoding either ZFR11-VP1-mCherry (LNP1, n = 9), VP1 alone (LNP2, n = 10), or PBS control (n = 6). Data are presented as individual animals with mean ± s.e.m. Statistical analysis was performed using one-way ANOVA. $^*P \leq 0.05$ was considered statistically significant. **(B)** Plasma concentrations of liver function biomarkers such as alanine aminotransferase (ALT) and total bile acids measured three weeks post-AAV2-mCherry challenge in the same animals. Data are presented as mean + s.e.m. Statistical analysis was performed using one-way ANOVA followed by Dunnett's multiple comparisons test versus PBS control. $^*P \leq 0.05$ was considered statistically significant.

**Table S1.** Target sequences and genomic positions of zinc finger repressors designed for *Myd88* promoter regulation. Sixteen ZFRs were designed to target specific 28 bp sequences within the *Myd88* promoter region. Target sequences are shown in 5' to 3' orientation with lowercase letters indicating flanking nucleotides and uppercase letters indicating the core binding site. Positions are designated relative to the transcription start site (TSS), with negative values indicating upstream locations. ZFRs are categorized by strand orientation: forward strand (ZF1-ZF8) and reverse strand (ZF9-ZF16). Extended positions refer to the expanded binding regions that include flanking sequences recognized by the zinc finger domains.

| ZFR ID | Target Sequence (5' → 3') | Positi | Stran | Distance from |
|---|---|---|---|---|
| ZF | ccGGATCCCACGCGGGGTCtcccg | Extend r210 | Rever | −210 bp upstream |
| Z] | ccACAGGCGAGCGTACTGGAcggc | Extend r135 | Forwa | −135 bp upstream |
| Z] | caCAGGCGAGCGTACTGGACggca | Extend r136 | Forwa | −136 bp upstream |
| Z] | gcGTACTGGACGGCACCGGGggcc | Extend r145 | Forwa | −145 bp upstream |
| Z] | ggACGGCACCGGGGGCCCAGggtt | Extend r152 | Forwa | −152 bp upstream |
| Z] | cgGCACCGGGGGCCCAGGGTtgcc | Extend r155 | Forwa | −155 bp upstream |
| Z] | ggCACCGGGGGCCCAGGGTTgcct | Extend r156 | Forwa | −156 bp upstream |
| Z] | acCGGGGGCCCAGGGTTGCCtgcc | Extend r159 | Forwa | −159 bp upstream |
| Z] | ccCCCGCGTGGGATCCGGGTccct | Extend r197 | Forwa | −197 bp upstream |
| Z] | ttGCTAGAATCTAGACTACGgggc | Extend r63a | Rever | −63 bp upstream |
| ZF | ccGGTGCCGTCCAGTACGCTcgcc | Extend r159 | Rever | −159 bp upstream |
| ZF | ccAGGGACCGGGATCCCACGcggg | Extend r218 | Rever | −218 bp upstream |
| ZF | aaGGAGTCCAGGGACCCGGAtccc | Extend r225 | Rever | −225 bp upstream |
| ZF | agGGTATGGAGAACATGAAGgagt | Extend r242 | Rever | −242 bp upstream |
| ZF | taAGCCGCACCAAGGGTATGgaga | Extend r254 | Rever | −254 bp upstream |
| ZF | acGTTAAGCGCGCACCAAGGGtat a | Extend r258 | Rever | −258 bp upstream |

**Table S2.** Physicochemical characterization of 306O$_{10}$ lipid nanoparticles. Size distribution, polydispersity index (PDI), and zeta potential were measured by dynamic light scattering. RNA entrapment efficiency was determined using Quant-iT™ RiboGreen™ RNA Assay. Ionization potential was measured using a 6-(p-toluidino)-2-naphthalenesulfonic acid sodium salt (TNS) assay to assess endosomal escape capability. Data are reported as means ± SEM, n=3.

| | **ZFR11-306O$_{10}$** | **VP1-306O$_{10}$** | **ZFR11- VP1-30[illegible]$_{10}$** |
|---|---|---|---|
| Size by number (n[illegible] | 103.4 ± 8.2 | 132.3 ± 13.3 | 123.6 ± 3.3 |
| PDI | 0.2158 ± 0.0136 | 0.2422 ± 0.0071 | 0.1572 ± 0.0071 |
| Zeta potential | -2.680 ± 0.71[illegible] | -4.441 ± 0.513 | -2.559 ± 0.66[illegible] |
| RNA entrapmen[illegible] efficiency (%) | 62.4 | 52.5 | 55.0 |
| Ionization potenti[illegible] (pH) | 6.76 | 6.16 | 6.08 |

**Supplementary Table 3.** Sequences of the zinc finger DNA fragments

| **DNA Fragment** | **Sequence** |
|---|---|
| ZF1 | ATGGACTACAAAGACCATGACGGTGATTATAAAGATCATGACATCGATTACAAGGA[illegible]<br>ACGATGACAAGATGGCCCCCAAGAAGAAGAGGAAGGTCGGCATTCATGGGGTACCCG<br>CCGCTATGGCTGAGAGGCCCTTCCAGTGTCGAATCTGCATGCGTAACTTCAGTCAGTC<br>CGGCCACCTGGCCCGCCACATCCGCACCCACACCGGCGAGAAGCCTTTTGCCTGTGA<br>CATTTGTGGGAGGAAATTTGCCAACGACTGGGACCGCCGCGTGCATACCAAGATACA<br>CACGGGCAGCCAAAAGCCCTTCCAGTGTCGAATCTGCATGCGTAACTTCAGTCACAA<br>GCAGCACCGCGACGCCCACATCCGCACCCACACCGGCGAGAAGCCTTTTGCCTGTGA<br>CATTTGTGGGAGGAAATTTGCCCGCTCCGCCAACCTGACCCGCCATACCAAGATACAC<br>ACGGGATCTCAGAAGCCCTTCCAGTGTCGAATCTGCATGCGTAACTTCAGTGACCGCT<br>CCCACCTGACCCGCCACATCCGCACCCACACCGGCGAGAAGCCTTTTGCCTGTGACA<br>TTTGTGGGAGGAAATTTGCCCAGTCCGGCACCCGCAAGACCCATACCAAGATACACCT<br>GCGCCAAAAAGATGCGGCCCGGGGATCC |
| ZF2 | ATGGACTACAAAGACCATGACGGTGATTATAAAGATCATGACATCGATTACAAGGA[illegible]<br>ACGATGACAAGATGGCCCCCAAGAAGAAGAGGAAGGTCGGCATTCATGGGGTACCCG<br>CCGCTATGGCTGAGAGGCCCTTCCAGTGTCGAATCTGCATGCGTAACTTCAGTGACCG<br>CTCCAACCTGTCCCGCCACATCCGCACCCACACCGGCGAGAAGCCTTTTGCCTGTGA<br>CATTTGTGGGAGGAAATTTGCCCGCACCGACGCCCGCCGCGGCCATACCAAGATACAC<br>ACGGGCAGCCAAAAGCCCTTCCAGTGTCGAATCTGCATGCGTAACTTCAGTCAGTCC<br>GGCGCCCTGGCCCGCCACATCCGCACCCACACCGGCGAGAAGCCTTTTGCCTGTGAC<br>ATTTGTGGGAGGAAATTTGCCATGGCCTGTTGTCGCTACGCCCATACCAAGATACACA<br>CGGGATCTCAGAAGCCCTTCCAGTGTCGAATCTGCATGCGTAACTTCAGTCGCTCCGA<br>CACCCTGTCCGAGCACATCCGCACCCACACCGGCGAGAAGCCTTTTGCCTGTGAC[illegible] |

| | |
|---|---|
| | TGTGGGAGGAAATTTGCCAAGCCGTACAACCTGCAGCAGCATACCAAGATACACC<br>CGCCAAAAAGATGCGGCCCGGGGATCC |
| ZF3 | ATGGACTACAAAGACCATGACGGTGATTATAAAGATCATGACATCGATTACAAGGA<br>ACGATGACAAGATGGCCCCCAAGAAGAAGAGGAAGGTCGGCATTCATGGGGTACCCG<br>CCGCTATGGCTGAGAGGCCCTTCCAGTGTCGAATCTGCATGCGTAACTTCAGTCGCTC<br>CGACCACCTGTCCCGCCACATCCGCACCCACACCGGCGAGAAGCCTTTTGCCTGTGA<br>CATTTGTGGGAGGAAATTTGCCGACCGCTCCGTGCTGGCCCGCCATACCAAGATACAC<br>ACGGGCAGCCAAAAGCCCTTCCAGTGTCGAATCTGCATGCGTAACTTCAGTGACCGC<br>TCCCACCTGACCCGCCACATCCGCACCCACACCGGCGAGAAGCCTTTTGCCTGTGAC<br>ATTTGTGGGAGGAAATTTGCCGACCGCTCCAACCTGACCCGCCATACCAAGATACACA<br>CGGGATCTCAGAAGCCCTTCCAGTGTCGAATCTGCATGCGTAACTTCAGTCGCTCCGA<br>CGTGCTGTCCGAGCACATCCGCACCCACACCGGCGAGAAGCCTTTTGCCTGTGACATT<br>TGTGGGAGGAAATTTGCCCAGTCCGGCTCCCTGACCCGCCATACCAAGATACACCTGC<br>GCCAAAAAGATGCGGCCCGGGGATCC |
| ZF4 | ATGGACTACAAAGACCATGACGGTGATTATAAAGATCATGACATCGATTACAAGGA<br>ACGATGACAAGATGGCCCCCAAGAAGAAGAGGAAGGTCGGCATTCATGGGGTACCCG<br>CCGCTATGGCTGAGAGGCCCTTCCAGTGTCGAATCTGCATGCGTAACTTCAGTCGTAG<br>TGACAACCTGAGCGAACACATCCGCACCCACACAGGCGAGAAGCCTTTTGCCTGTGA<br>CATTTGTGGGAGGAAATTTGCCGCCAGCAAGACCCGCAAAAACCATACCAAGATACA<br>CACGGGCAGCCAAAAGCCCTTCCAGTGTCGAATCTGCATGCGTAACTTCAGTCGCTCC<br>GACCACCTGTCCCGCCACATCCGCACCCACACCGGCGAGAAGCCTTTTGCCTGTGAC<br>ATTTGTGGGAGGAAATTTGCCCAGCAGTGGGACCGCAAGCAGCATACCAAGATACAC<br>ACGGGATCTCAGAAGCCCTTCCAGTGTCGAATCTGCATGCGTAACTTCAGTCAGTCCG<br>GCGACCTGACCCGCCACATCCGCACCCACACCGGCGAGAAGCCTTTTGCCTGTGACA<br>TTTGTGGGAGGAAATTTGCCCGCAACGACATCCTGGCCTCCCATACCAAGATACACCT<br>GCGCCAAAAAGATGCGGCCCGGGGATCC |
| ZF5 | ATGGACTACAAAGACCATGACGGTGATTATAAAGATCATGACATCGATTACAAGGA<br>ACGATGACAAGATGGCCCCCAAGAAGAAGAGGAAGGTCGGCATTCATGGGGTACCCG<br>CCGCTATGGCTGAGAGGCCCTTCCAGTGTCGAATCTGCATGCGTAACTTCAGTCTGCG<br>CCACCACCTGACCCGCCACATCCGCACCCACACCGGCGAGAAGCCTTTTGCCTGTGA<br>CATTTGTGGGAGGAAATTTGCCCGCCGCGACTGGCGCCGCGACCATACCAAGATACAC<br>ACGGGCAGCCAAAAGCCCTTCCAGTGTCGAATCTGCATGCGTAACTTCAGTGAGCGC<br>GGCACCCTGGCCCGCCACATCCGCACCCACACCGGCGAGAAGCCTTTTGCCTGTGAC<br>ATTTGTGGGAGGAAATTTGCCCGCTCCGACCACCTGTCCCGCCATACCAAGATACACA<br>CGGGATCTCAGAAGCCCTTCCAGTGTCGAATCTGCATGCGTAACTTCAGTCGCTCCGA<br>CACCCTGTCCGAGCACATCCGCACCCACACCGGCGAGAAGCCTTTTGCCTGTGACATT<br>TGTGGGAGGAAATTTGCCCAGTCCGGCGACCTGACCCGCCATACCAAGATACACCTG<br>CGCCAAAAAGATGCGGCCCGGGGATCC |
| ZF6 | ATGGACTACAAAGACCATGACGGTGATTATAAAGATCATGACATCGATTACAAGGA<br>ACGATGACAAGATGGCCCCCAAGAAGAAGAGGAAGGTCGGCATTCATGGGGTACCCG<br>CCGCTATGGCTGAGAGGCCCTTCCAGTGTCGAATCTGCATGCGTAACTTCAGTACCTC<br>CGGCTCCCTGTCCCGCCACATCCGCACCCACACCGGCGAGAAGCCTTTTGCCTGTGA<br>CATTTGTGGGAGGAAATTTGCCCGCTCCGACCACCTGACCCAGCATACCAAGATACAC<br>ACGGGCAGCCAAAAGCCCTTCCAGTGTCGAATCTGCATGCGTAACTTCAGTGACCGC<br>TCCGACCTGTCCCGCCACATCCGCACCCACACCGGCGAGAAGCCTTTTGCCTGTGAC<br>ATTTGTGGGAGGAAATTTGCCCGCTCCACCCACCTGGTGCGCCATACCAAGATACACA<br>CGGGATCTCAGAAGCCCTTCCAGTGTCGAATCTGCATGCGTAACTTCAGTCGCTCCGA<br>CCACCTGTCCGAGCACATCCGCACCCACACCGGCGAGAAGCCTTTTGCCTGTGACATT<br>TGTGGGAGGAAATTTGCCGACCGCTCCAACCGCAAGACCCATACCAAGATACACCTG<br>CGCCAAAAAGATGCGGCCCGGGGATCC |
| | ATGGACTACAAAGACCATGACGGTGATTATAAAGATCATGACATCGATTACAAGGA<br>ACGATGACAAGATGGCCCCCAAGAAGAAGAGGAAGGTCGGCATTCATGGGGTACCCG<br>CCGCTATGGCTGAGAGGCCCTTCCAGTGTCGAATCTGCATGCGTAACTTCAGTGAGCG<br>CGGCACCCTGGCCCGCCACATCCGCACCCACACCGGCGAGAAGCCTTTTGCCTGT |

| | |
|---|---|
| ZF7 | CATTTGTGGGAGGAAATTTGCCACCTCCGGCTCCCTGACCCGCCATACCAAGATAC<br>ACGGGCAGCCAAAAGCCCTTCCAGTGTCGAATCTGCATGCGTAACTTCAGTCGTAGTG<br>ACCACCTGAGCAACCACATCCGCACCCACACAGGCGAGAAGCCTTTTGCCTGTGACA<br>TTTGTGGGAGGAAATTTGCCGACAACAGGGACCGCATAAAGCATACCAAGATACACA<br>CGGGATCTCAGAAGCCCTTCCAGTGTCGAATCTGCATGCGTAACTTCAGTCGTAGTGA<br>CCACCTGAGCAACCACATCCGCACCCACACAGGCGAGAAGCCTTTTGCCTGTGACAT<br>TTGTGGGAGGAAATTTGCCCGGAGCGACGACCGCAAAAACCATACCAAGATACACCT<br>GCGCCAAAAAGATGCGGCCCGGGGATCC |
| ZF8 | ATGGACTACAAAGACCATGACGGTGATTATAAAGATCATGACATCGATTACAAGGA<br>ACGATGACAAGATGGCCCCCAAGAAGAAGAGGAAGGTCGGCATTCATGGGGTACCCG<br>CCGCTATGGCTGAGAGGCCCTTCCAGTGTCGAATCTGCATGCGTAACTTCAGTCTGCG<br>CCACCACCTGACCCGCCACATCCGCACCCACACCGGCGAGAAGCCTTTTGCCTGTGA<br>CATTTGTGGGAGGAAATTTGCCCGCCGCGACACCCTGCTGGACCATACCAAGATACAC<br>ACGGGCAGCCAAAAGCCCTTCCAGTGTCGAATCTGCATGCGTAACTTCAGTACCTCCG<br>GCAACCTGACCCGCCACATCCGCACCCACACCGGCGAGAAGCCTTTTGCCTGTGACA<br>TTTGTGGGAGGAAATTTGCCCGCCGCTACTACCTGCGCCTGCATACCAAGATACACAC<br>GGGATCTCAGAAGCCCTTCCAGTGTCGAATCTGCATGCGTAACTTCAGTCGCTCCGAC<br>GACCTGACCCGCCACATCCGCACCCACACCGGCGAGAAGCCTTTTGCCTGTGACATTT<br>GTGGGAGGAAATTTGCCCACCGCCGCTCCCGCGACCAGCATACCAAGATACACCTGC<br>GCCAAAAAGATGCGGCCCGGGGATCC |
| ZF9 | ATGGACTACAAAGACCATGACGGTGATTATAAAGATCATGACATCGATTACAAGGA<br>ACGATGACAAGATGGCCCCCAAGAAGAAGAGGAAGGTCGGCATCCACGGGGTACCC<br>GCCGCTATGGCTGAGAGGCCCTTCCAGTGTCGAATCTGCATGCGTAACTTCAGTCGTA<br>GTGACACCCTGAGCGCACACATCCGCACCCACACAGGCGAGAAGCCTTTTGCCTGTG<br>ACATTTGTGGGAGGAAATTTGCCGCCAACAGCACCCGCACAAACCATACCAAGATAC<br>ACACGGGCAGCCAAAAGCCCTTCCAGTGTCGAATCTGCATGCGTAACTTCAGTCGCT<br>CCGACAACCTGTCCACCCACATCCGCACCCACACCGGCGAGAAGCCTTTTGCCTGTG<br>ACATTTGTGGGAGGAAATTTGCCGACCGCTCCGCCCTGGCCCGCCATACCAAGATACA<br>CACGGGATCTCAGAAGCCCTTCCAGTGTCGAATCTGCATGCGTAACTTCAGTCAGTCC<br>GCCCACCGCAAGAACCACATCCGCACCCACACCGGCGAGAAGCCTTTTGCCTGTGAC<br>ATTTGTGGGAGGAAATTTGCCCTGAAGCAGGTGCTGGTGCGCCATACCAAGATACACC<br>TGCGCCAAAAAGATGCGGCCCGGGGATCC |
| ZF1 | ATGGACTACAAAGACCATGACGGTGATTATAAAGATCATGACATCGATTACAAGGA<br>ACGATGACAAGATGGCCCCCAAGAAGAAGAGGAAGGTCGGCATCCACGGGGTACCC<br>GCCGCTATGGCTGAGAGGCCCTTCCAGTGTCGAATCTGCATGCGTAACTTCAGTCAGT<br>CCTCCGACCTGTCCCGCCACATCCGCACCCACACCGGCGAGAAGCCTTTTGCCTGTG<br>ACATTTGTGGGAGGAAATTTGCCGACGCCGGCAACCGCAACAAGCATACCAAGATAC<br>ACACGGGCAGCCAAAAGCCCTTCCAGTGTCGAATCTGCATGCGTAACTTCAGTCGCT<br>CCGACAACCTGTCCACCCACATCCGCACCCACACCGGCGAGAAGCCTTTTGCCTGTG<br>ACATTTGTGGGAGGAAATTTGCCGACAACTCCTACCTGCCGCGCCATACCAAGATACA<br>CACGGGATCTCAGAAGCCCTTCCAGTGTCGAATCTGCATGCGTAACTTCAGTGAGCGC<br>GGCACCCTGGCCCGCCACATCCGCACCCACACCGGCGAGAAGCCTTTTGCCTGTGAC<br>ATTTGTGGGAGGAAATTTGCCACCTCCGGCTCCCTGACCCGCCATACCAAGATACACC<br>TGCGCCAAAAAGATGCGGCCCGGGGATCC |
| ZF1 | ATGGACTACAAAGACCATGACGGTGATTATAAAGATCATGACATCGATTACAAGGA<br>ACGATGACAAGATGGCCCCCAAGAAGAAGAGGAAGGTCGGCATCCACGGGGTACCC<br>GCCGCTATGGCTGAGAGGCCCTTCCAGTGTCGAATCTGCATGCGTAACTTCAGTGACC<br>GCTCCGCCCTGTCCCGCCACATCCGCACCCACACCGGCGAGAAGCCTTTTGCCTGTG<br>ACATTTGTGGGAGGAAATTTGCCCGCTCCGACCACCTGTCCCGCCATACCAAGATACA<br>CACGGGCAGCCAAAAGCCCTTCCAGTGTCGAATCTGCATGCGTAACTTCAGTCGCTCC<br>GACGACCTGACCCGCCACATCCGCACCCACACCGGCGAGAAGCCTTTTGCCTGTGAC<br>ATTTGTGGGAGGAAATTTGCCGACCGCTCCAACCTGAAGGCCCATACCAAGATACACA<br>CGGGATCTCAGAAGCCCTTCCAGTGTCGAATCTGCATGCGTAACTTCAGTGACTCCTC<br>CGACCGCAAGAAGCACATCCGCACCCACACCGGCGAGAAGCCTTTTGCCTGTGAC |

| | |
|---|---|
| | TTGTGGGAGGAAATTTGCCCAGTCCGGCCACCTGTCCCGCCATACCAAGATACA[illegible]<br>CGCCAAAAAGATGCGGCCCGGGGATCC |
| ZI | ATGGACTACAAAGACCATGACGGTGATTATAAAGATCATGACATCGATTACAAGG<br>ACGATGACAAGATGGCCCCCAAGAAGAAGAGGAAGGTCGGCATCCACGGGGTACCC<br>GCCGCTATGGCTGAGAGGCCCTTCCAGTGTCGAATCTGCATGCGTAACTTCAGTCGTA<br>GTGACACCCTGAGCGCACACATCCGCACCCACACAGGCGAGAAGCCTTTTGCCTGTG<br>ACATTTGTGGGAGGAAATTTGCCGACAAGAGGACCCGCACAACCCATACCAAGATAC<br>ACACGGGCAGCCAAAAGCCCTTCCAGTGTCGAATCTGCATGCGTAACTTCAGTACCTC<br>CGGCAACCTGACCCGCCACATCCGCACCCACACCGGCGAGAAGCCTTTTGCCTGTGA<br>CATTTGTGGGAGGAAATTTGCCCGCGAGGGCGACCTGAAGCAGCATACCAAGATACA<br>CACGGGATCTCAGAAGCCCTTCCAGTGTCGAATCTGCATGCGTAACTTCAGTGACCGC<br>TCCAACCTGTCCCGCCACATCCGCACCCACACCGGCGAGAAGCCTTTTGCCTGTGAC<br>ATTTGTGGGAGGAAATTTGCCCGCTCCGACCACCTGACCCAGCATACCAAGATACACC<br>TGCGCCAAAAAGATGCGGCCCGGGGATCC |
| ZI | ATGGACTACAAAGACCATGACGGTGATTATAAAGATCATGACATCGATTACAAGG<br>ACGATGACAAGATGGCCCCCAAGAAGAAGAGGAAGGTCGGCATCCACGGGGTACCC<br>GCCGCTATGGCTGAGAGGCCCTTCCAGTGTCGAATCTGCATGCGTAACTTCAGTCAGT<br>CCGGCCACCTGGCCCGCCACATCCGCACCCACACCGGCGAGAAGCCTTTTGCCTGTG<br>ACATTTGTGGGAGGAAATTTGCCGACAAGCGCGACCGCCGCACCCATACCAAGATAC<br>ACACGGGCAGCCAAAAGCCCTTCCAGTGTCGAATCTGCATGCGTAACTTCAGTCAGT<br>CCTCCCACCTGACCCGCCACATCCGCACCCACACCGGCGAGAAGCCTTTTGCCTGTG<br>ACATTTGTGGGAGGAAATTTGCCCGCCTGGACAACCGCACCGCCCATACCAAGATACA<br>CACGGGATCTCAGAAGCCCTTCCAGTGTCGAATCTGCATGCGTAACTTCAGTGACCGC<br>TCCGCCCGCACCCGCCACATCCGCACCCACACCGGCGAGAAGCCTTTTGCCTGTGAC<br>ATTTGTGGGAGGAAATTTGCCCAGTCCGGCCACCTGTCCCGCCATACCAAGATACACC<br>TGCGCCAAAAAGATGCGGCCCGGGGATCC |
| ZI | ATGGACTACAAAGACCATGACGGTGATTATAAAGATCATGACATCGATTACAAGG<br>ACGATGACAAGATGGCCCCCAAGAAGAAGAGGAAGGTCGGCATCCACGGGGTACCC<br>GCCGCTATGGCTGAGAGGCCCTTCCAGTGTCGAATCTGCATGCGTAACTTCAGTCGCT<br>CCGACAACCTGTCCGAGCACATCCGCACCCACACCGGCGAGAAGCCTTTTGCCTGTG<br>ACATTTGTGGGAGGAAATTTGCCGTGCGCCGCGCCCTGTCCTCCCATACCAAGATACA<br>CACGGGCAGCCAAAAGCCCTTCCAGTGTCGAATCTGCATGCGTAACTTCAGTGACCA<br>GTCCAACCTGCGCGCCCACATCCGCACCCACACCGGCGAGAAGCCTTTTGCCTGTGA<br>CATTTGTGGGAGGAAATTTGCCAAGCAGTACCGCCTGATCACCCATACCAAGATACAC<br>ACGGGATCTCAGAAGCCCTTCCAGTGTCGAATCTGCATGCGTAACTTCAGTCGCTCCG<br>ACACCCTGTCCCAGCACATCCGCACCCACACCGGCGAGAAGCCTTTTGCCTGTGACA<br>TTTGTGGGAGGAAATTTGCCACCTCCGGCCACCTGTCCCGCCATACCAAGATACACCT<br>GCGCCAAAAAGATGCGGCCCGGGGATCC |
| ZI | ATGGACTACAAAGACCATGACGGTGATTATAAAGATCATGACATCGATTACAAGG<br>ACGATGACAAGATGGCCCCCAAGAAGAAGAGGAAGGTCGGCATCCACGGGGTACCC<br>GCCGCTATGGCTGAGAGGCCCTTCCAGTGTCGAATCTGCATGCGTAACTTCAGTCGCT<br>CCGACACCCTGTCCCAGCACATCCGCACCCACACCGGCGAGAAGCCTTTTGCCTGTG<br>ACATTTGTGGGAGGAAATTTGCCACCTCCGGCCACCTGTCCCGCCATACCAAGATACA<br>CACGGGCAGCCAAAAGCCCTTCCAGTGTCGAATCTGCATGCGTAACTTCAGTCGTAGT<br>GACACCCTGAGCGTACACATCCGCACCCACACAGGCGAGAAGCCTTTTGCCTGTGAC<br>ATTTGTGGGAGGAAATTTGCCGACAACAGCACCCGCATAAAGCATACCAAGATACAC<br>ACGGGATCTCAGAAGCCCTTCCAGTGTCGAATCTGCATGCGTAACTTCAGTCGCTCCG<br>ACGACCTGACCCGCCACATCCGCACCCACACCGGCGAGAAGCCTTTTGCCTGTGACA<br>TTTGTGGGAGGAAATTTGCCATGTCCTACCAGCTGGGCCGCCATACCAAGATACACCT<br>GCGCCAAAAAGATGCGGCCCGGGGATCC |
| ZI | ATGGACTACAAAGACCATGACGGTGATTATAAAGATCATGACATCGATTACAAGG<br>ACGATGACAAGATGGCCCCCAAGAAGAAGAGGAAGGTCGGCATCCACGGGGTACCC<br>GCCGCTATGGCTGAGAGGCCCTTCCAGTGTCGAATCTGCATGCGTAACTTCAGTCGTA<br>GTGACCACCTGAGCCGGCACATCCGCACCCACACAGGCGAGAAGCCTTTTGCC[illegible] |

| | |
|---|---|
| | ACATTTGTGGGAGGAAATTTGCCGACAGGAGCAACCGCAAAACCCATACCAAG<br>ACACGGGCAGCCAAAAGCCCTTCCAGTGTCGAATCTGCATGCGTAACTTCAGTGACC<br>GCTCCAACCTGTCCCGCCACATCCGCACCCACACCGGCGAGAAGCCTTTTGCCTGTG<br>ACATTTGTGGGAGGAAATTTGCCAACTCCCGCAACCTGCGCAACCATACCAAGATACA<br>CACGGGATCTCAGAAGCCCTTCCAGTGTCGAATCTGCATGCGTAACTTCAGTCGCTCC<br>GACAACCTGTCCGTGCACATCCGCACCCACACCGGCGAGAAGCCTTTTGCCTGTGAC<br>ATTTGTGGGAGGAAATTTGCCACCTCCGGCTCCCTGACCCGCCATACCAAGATACACC<br>TGCGCCAAAAAGATGCGGCCCGGGGATCC |
| ZF<br>HP1a-Krab | **ATG**GACTACAAAGACCATGACGGTGATTATAAAGATCATGACATCGATTACAA<br>TGACGATGACAAGATGGCCCCCAAGAAGAAGAGGAAGGTCGGCATCCACGGGGTAC<br>CCGCCGCTATGGCTGAGAGGCCCTTCCAGTGTCGAATCTGCATGCGTAACTTCAGTG<br>ACCGCTCCGCCCTGTCCCGCCACATCCGCACCCACACCGGCGAGAAGCCTTTTGCCT<br>GTGACATTTGTGGGAGGAAATTTGCCCGCTCCGACCACCTGTCCCGCCATACCAAGA<br>TACACACGGGCAGCCAAAAGCCCTTCCAGTGTCGAATCTGCATGCGTAACTTCAGTC<br>GCTCCGACGACCTGACCCGCCACATCCGCACCCACACCGGCGAGAAGCCTTTTGCCT<br>GTGACATTTGTGGGAGGAAATTTGCCGACCGCTCCAACCTGAAGGCCCATACCAAGA<br>TACACACGGGATCTCAGAAGCCCTTCCAGTGTCGAATCTGCATGCGTAACTTCAGTG<br>ACTCCTCCGACCGCAAGAAGCACATCCGCACCCACACCGGCGAGAAGCCTTTTGCCT<br>GTGACATTTGTGGGAGGAAATTTGCCCAGTCCGGCCACCTGTCCCGCCATACCAAGA<br>TACACCTGCGCCAAAAAGATGCGGCCCGGGGATCCCCATGGCCCAAGAAGAAGAGG<br>AAGGTGAGTGGTGGAGGAAGTGGCGGGTCAGGGTCGAGCCCCAAGAAAAAACGGA<br>AAGTGGAGGCATCAATGAAGGAGGGAGAAAACAACAAACCCAGGGAGAAAAGTGA<br>AGGGAATAAGAGAAAAAGCTCCTTCTCTAACAGTGCAGACGATATCAAGTCCAAGA<br>AAAAGCGGGAGCAGTCTAATGACATTGCTAGGGGCTTCGAGAGAGGACTGGAGCCA<br>GAAAAAATCATTGGGGCAACCGACAGCTGCGGCGATCTGATGTTTCTCATGAAATGG<br>AAGGACACAGATGAGGCCGACCTGGTGCTCGCCAAAGAAGCTAACGTGAAGTGTCC<br>CCAGATCGTCATTGCTTTTTACGAGGAAAGGCTCACCTGGCACGCATATCCTGAGGA<br>TGCCGAAAACAAGGAGAAGGAATCAGCTAAGAGCTCGGGAGGTGGTTCGGGTGGCT<br>CTGGATCAATGGACGCGAAATCACTTACGGCATGGTCGAGAACACTGGTTACGTTCA<br>AGGACGTGTTTGTGGACTTTACACGTGAGGAGTGGAAATTGCTGGATACTGCGCAAC<br>AAATTGTGTATCGAAATGTCATGCTTGAGAATTACAAGAACCTCGTCAGTCTCGGAT<br>ACCAGTTGACGAAACCGGATGTGATCCTTAGGCTCGAAAAGGGGGAAGAACCTTGG<br>CTGGTA**TAG** |

**Table S4.** Mouse qPCR primers - 5’ to 3’

| | |
|---|---|
| Myd88 primer FW | GTGAGGATATACTGAAGGAGCT |
| Myd88 primer RV | CTGTAAAGGCTTCTCGGACTC |
| GFP-FW | CAACCACTACCTGAGCACCC |
| GFP-RV | GTCCATGCCGAGAGTGATCC |
| mCherry-FW | GCGCGTGATGAACTTCGAGG |
| mCherry-RV | CCGTCCTTCAGCTTCAGCCT |
| Stat4-FW | TCAGTGAGAGCCATCTTGGAGC |
| Stat4-RV | TGTAGTCTCGCAGGATGTCAGC |
| IFNa-FW | GGACT T TGGAT TCCCGCAGGA |
| IFNa-RV | GCTGCATCAGACAGCCT TGCAC |
| IFN-γ-PRIMETIME | IDT:MM.PT.58.41769240 |

| | |
|---|---|
| IFN-β-PRIMETIME | IDT:MM.PT.58.30132453.g |
| Icam-1-FW | CAATTTCTCATGCCGCACAG |
| Icam-1-RV | AGCTGGAAGATCGAAAGTCCG |
| TNFa-FW | AGGCTGCCCCGACTACGT |
| TNFa-RV | GACTTTCTCCTGGTATGAGATA |
| NCF-FW | GCTGCGTGAACACTATCCTGG |
| NCF-RV | AGGTCGTACTTCTCCATTCTGT[illegible] |
| 18S-FW | GAGACTCTGGCATGCTAACTA |
| 18S-RV | GGACATCTAAGGGCATCACAG |
| IL6-PRIMETIME | IDT: Mm.PT.58.100 |
| IL1B-FW | TCGCTCAGGGTCACAAGAAA |
| IL1B-RV | CATCAGAGGCAAGGAGGAAAAC |
